# Empowerment interventions designed for persons living with chronic disease - a systematic review and meta-analysis of the components and efficacy of format on patient-reported outcomes

**DOI:** 10.1186/s12913-023-09895-6

**Published:** 2023-08-25

**Authors:** Natalie Stepanian, Marie Hamilton Larsen, Joshua B. Mendelsohn, Kari L. Mariussen, Kristin Heggdal

**Affiliations:** 1 College of Health Professions, Pace University, One Pace Plaza, New York, NY 10038 USA; 2 Lovisenberg Diaconal University College, Lovisenberggaten 15, 0456 Oslo, Norway; 3 Faculty of Health, VID Specialized University, Theodor Dahls Vei 10, 0370 Oslo, Norway

**Keywords:** Systematic review, Meta-analysis, Empowerment, Self-management, Interventions, Person-centered care, Chronic disease

## Abstract

**Background:**

Empowerment approaches are essential for building the capacity of individuals with chronic disease to be in control of their health. Reviews of empowerment interventions have been focused on specific chronic diseases, thereby limiting the scope of findings. This study had three aims: 1) to describe the characteristics of empowerment interventions covering a broad range of chronic diseases, 2) to clarify consistency with the World Health Organization`s (WHO) definition of empowerment as a process composed of four fundamental components and 3) to summarize outcome measures and estimate the effects in group and individual intervention formats.

**Methods:**

Systematic literature review and meta-analysis. CINAHL, Medline, Embase, PsycINFO, Web of Science, COCHRANE and Central Register of Controlled Trials were searched using Chronic Disease, NCD, Empowerment, as MeSH terms. Eligible randomized and quasi randomized controlled trials were included. Review Manager 5.4 was used to conduct the meta-analysis. Risk of bias was assessed with the Cochrane risk-of-bias tool (ROB 2).

**Results:**

Thirty-nine articles representing 8,011 participants were included in the review. A majority (82%) of studies reported robust evidence for changes on study-defined outcome measures in favor of interventions. Intervention content was assessed against WHO’s four fundamental components of empowerment, showing that all studies incorporated one component, but none targeted all components. Components reflecting knowledge acquisition, patient engagement with their health care providers and facilitating environment were scarcely reported. Meta-analyses found evidence for positive effects of group-format interventions measuring empowerment, HbA1c, and self-efficacy. Effects on empowerment were also found in some individual-format interventions. High levels of heterogeneity and variability among the conceptual frameworks were identified.

**Conclusion:**

Empowerment interventions in group-format were most efficient, however, considerable conceptual inconsistencies were identified. Future studies should consolidate conceptual understandings by using WHO’s empowerment framework to ensure that fundamental components of empowerment are explicitly included in intervention design. Furthermore, there is a need to clarify the role of empowerment through pathways that include patient activation, self- management, and clinical outcomes. This systematic review will inform the clinicians and researchers who aim to develop novel empowerment interventions to assist patients in the process of gaining control of their health.

**Trial registration:**

PROSPERO: International Prospective register of systematic reviews ID=CRD42020178286.

**Supplementary Information:**

The online version contains supplementary material available at 10.1186/s12913-023-09895-6.

## Background

The World Health Organization (WHO) has targeted a reduction of 30% in the number of premature deaths due to chronic disease by 2030 [1] and highlighted the importance of patient empowerment in prevention and health promotion efforts. The main idea is to redistribute power from the health professionals to the patients who handle the challenges of chronic disease on a daily basis [2].

Within healthcare, the empowerment approach is a patient-centric, collaborative approach that starts with the principle of individuals’ inherent capacity to be in control of one’s own life. It has been described as a complex experience of personal change, facilitated by healthcare providers [3]. A major concern of people diagnosed with chronic disease is the multitudes of uncertainties they face, and the restrictions of their life space they encounter due to their health condition [4, 5]. This uncertainty may be accompanied by the experience of lack of control and the feeling of powerlessness that is connected to the disruption of the physical, psychological and social aspects of the patients’ lives [6–8]. Patients frequently undergo long periods of multimodal treatment and are challenged to change their lifestyle in order to prevent their chronic condition from worsening. However, patients possess internal and external strengths and self-management strategies to fight against the threat of deterioration while living with chronic disease [9]. These capacities function to empower patients to regain control through a process of health-related change [5, 10]. According to Castro, Regenmortel, et al. [11] “patients have come to be seen as experts of their own bodies, symptoms and situations, and patients’ experiential knowledge is now considered to be complementary to professional knowledge and important for the success of their treatment, self-care management, and for improving the quality of care” (p.1924). The challenge is to effectively utilize patient empowerment resources [6] and capabilities to promote health and wellness within chronic disease. Empowerment is an essential concept in this regard. A descriptive review [12] of 49 empirical studies showed that 35 different definitions were used to define empowerment and that the use of the concept has been inconsistent. Furthermore 38 different instruments were applied to measure empowerment. The lack of clear conceptualization of empowerment as well as the interchangeably use of empowerment with other related concepts, suggests the need to distinguish between empowerment as a psychosocial and health-related process, and self-management, self-efficacy, patient activation, health literacy, behavioral change and quality of life as indicators or outcomes of empowerment [12–14]. In this review, we have chosen to rely on WHO’s definition of the empowerment process as this implies a broad understanding of empowerment that allows for the inclusion of different definitions and interventions covering a variety of chronic disease diagnostic categories. WHO has defined four fundamental components of the empowerment process: patient participation, patient knowledge, patient skills, and the creation of a facilitating environment and have integrated empowerment in their guidelines for reaching sustainable goals [15–17]. A facilitating environment implies being listened to with regard to one’s concern, being engaged in shared decision-making with health care professionals and having access to high quality organized care, i.e. interventions for knowledge improvement and psychological support [18]. The WHO has published a handbook in 2021 [17] that focuses on empowerment and reaffirms the importance of social participation as fundamental for empowerment.

A wide variety of interventions that facilitate partnerships between patients and healthcare professionals (HCP) using a wide-range of approaches including patient education, shared decision-making, goal-setting, on self-evaluation, and motivational interviewing have been developed [19–21]. Cardoso Barbosa, de Queiroz Oliveira, et al. [22] integrative review showed that empowerment interventions have the potential to strengthen patient autonomy and the trust of individuals in shared decision-making, helping them to develop coping and communication skills, and implementing behavior changes related to their disease, underscoring the importance of focus on empowerment in interventions for living a good life with a chronic condition.

The focus of our review is to study empowerment interventions for patients with chronic disease across diagnostic categories. In a prior review, Chen and I-Chuan [23] demonstrated that empowerment-focused interventions improved the health status, psychological condition and quality of life (QOL) of chronically ill patients. Werbrouk, Swinnen, et al. [3]’s systematic review covering 2007–2017 included 32 randomized-controlled trials (RCT) of which 23 were included in a meta-analysis that estimated an overall interventional effect but with high heterogeneity. Samoocha, Bruinvels, et al. [24] studied the effect of web-based interventions for patient empowerment. The included Web-based interventions had a positive effect on empowerment in diabetes patients, on self-efficacy, however when compared to face-to-face delivery interventions, no significant effects were found for mastery. This 2010 review has not been updated, however, more recent systematic reviews involving patients with specific chronic conditions shows similar results. For example, a systematic review on web-based interventions targeting cardiovascular risk factors in older adults [25], found a potential to improve the cardiovascular risk profile, however, the effects were modest and declined with time. Another review assessing the effectiveness of internet empowerment-based self-management interventions within metabolic diseases [26] showed more positive results. These interventions significantly improved the health status of adults with metabolic diseases, in particular their exercise habits, HbA1c levels, body weight, empowerment and quality of life. The majority of studies were limited to patients with diabetes and had methodological issues with increasing risk of bias. When searching the Cochrane review database for empowerment interventions, we found that the majority of former reviews were disease-specific [27, 28]. Two reviews of interventions in chronic illness in general were found, however, these reviews did not mention empowerment. De Jongh, Gurol‐Urganci, et al. [29] reviewed interventions using mobile phone messages to facilitate self-management and found no statistical difference in health outcomes from text messages compared with usual care, however, moderate improvement in self-management capacity was found in diabetes patients. Smith, Wallace, et al. [30] reviewed the effectiveness of interventions designed to improve clinical and mental health outcomes and patient reported outcomes in people with multimorbidity in primary care and community settings and found no clear positive improvements, however, modest improvement was found in health outcomes among patients with depression.

The results from former reviews show mixed results while the efficacy of empowerment interventions for patients with different kinds of diagnosis, remains unclear. As former research has identified a wide variety of conceptual understandings of empowerment, there is a need to clarify whether interventions capture essential components of empowerment as described by WHO [15]. In response, our objective was to conduct a systematic review of empowerment interventions that covered a broad range of chronic diseases in order to assess if fundamental components of empowerment were included and to study intervention efficacy across diagnoses.

## Method

This systematic review and meta-analysis aimed to describe the characteristics of empowerment interventions covering a broad range of chronic diseases by a) summarizing intervention setting, structure, modality, content, and clarify consistency with WHO’s empowerment definition, b) to summarize outcome measures and estimate the effect in group and individual formats.

The study was designed as a systematic literature review and meta-analysis covering January 1^st^ 2016-March 25^th^ 2020 and reported according to PRISMA guidelines [31]. The protocol was published in PROSPERO (CRD42020178286).

### Search methods

The research question was structured according to PICOs (Population, Intervention, Comparison, Outcome, and Study design) (Table 1). Eligibility criteria was: a) scientific publication of original research, b) RCT/quasi experiments and c) included patients with different chronic diseases.

**Table 1:** Inclusion and exclusion criteria in PICOS (Population, Intervention, Comparison, Outcome, Study Design) format

	Inclusion criteria	Exclusion criteria
P (Population)	Chronic disease and/or noncommunicable diseases as defined by WHO	Children and adolescents < 18 years. Patients in active cancer treatment. People with severe mental illness.
I (Intervention)	Empowerment-based interventions or programs aiming to improve health-related outcomes. Interventions delivered inpatient, outpatient, or community settings related to patients with chronic disease(s)	Studies where empowerment was not the specific aim nor mentioned in the abstract
C (Comparison)	Control groups with no treatment or standard care control groups or attention control groups (participants receive some other attention) or waitlist	Studies without control-groups (pre/post not included)
O (Outcomes)	Patient-reported empowerment outcomes, health care outcomes and / or clinical outcomes related to empowerment like quality of life, self-management or health literacy. Clinical outcomes such as blood pressure or HbA1c	Outcomes not related to empowerment
S (Study design)	Experimental studies, defined as randomized controlled trials or quasi-experimental trials (e.g. non-randomized, pre-posttest)	Qualitative studies, cross-sectional studies, case–control studies, cohort studies and mixed methods design. Any type of systematic or non-systematic review, non-peer reviewed articles, grey literature, master and PhD thesis, conference proceedings, comments or opinion articles, official guidelines, editorials, and abstracts

A systematic search strategy was implemented in MEDLINE, Embase, PsycINFO, CINAHL, Web of Science, and COCHRANE Central Register of Controlled Trials (Supplementary file 1) and was reviewed using the Peer Review of Electronic Search Strategies (PRESS) checklist [32]. Controlled vocabulary (e.g., Medical Subject Headings in MEDLINE) and additional keywords were used to identify relevant search terms, and the RCT filter from Ovid Expert searches was applied from which time the prior systematic review by Chen and I-Chuan [23] had been completed. Reports written in English and Scandinavian languages were included.

### Search outcomes

Search results were imported to an EndNote library, and duplicates were removed. Reports were randomly divided in two halves and imported into Rayyan software for review by two teams of reviewers (X & X, X & X). The abstracts and titles were independently screened by two reviewers in accordance with the eligibility criteria. The two reviewers then retrieved and screened the full texts of the relevant studies, then reviewer pairs evaluated the alternate set of reports from the first screening. Disagreements about inclusion were solved by discussing with the whole team. The second screening resulted in 83 studies with significant differences in scope regarding interventions, populations and outcomes. In order to be able to do an in-depth description of the interventions, a pragmatic decision was made to exclude studies published prior to 2016. This choice resulted in the exclusion of 44 publications, of which 57% described patients with diabetes. Reviews covering empowerment interventions in diabetes are previously published [33–35]. A PRISMA flow chart was used to document the number of excluded studies and the reasons for exclusion.

### Quality appraisal

We applied the updated Cochrane Risk of Bias Assessment tool (ROB2). Reports were classified as having a low risk of bias, some concerns, or a high risk of bias [36]. All quality assessments were independently conducted in the review pairs and consensus was achieved through discussion among the whole team.

### Data extraction

The following data was extracted: author, year of publication, setting, patient diagnosis, type of health care professional delivering the intervention, modality, design, outcome measures and results (Table 2). Extracted data were cross-checked and finalized by all team members.

**Table 2:** Summary of individual studies

First author/ year/country	Aim	Design and theoretical framework	Study population and setting	Description of the intervention and control	Primary (1)/Secondary (2) prom outcome	Results related to the systematic review
Admiraal, et al. (2017) [37] Netherlands	To examine the effectiveness of a web-based tailored psychoeducational program (ENCOURAGE) for breast cancer patients, to empower patients to take control over prevailing problems	Randomized Controlled Trial (RCT) **Theory:** The theory of problem solving (Dunker, 1935)	*N* = 138 adult female breast cancer patients who recently completed neo-adjuvant chemotherapy. **Study group** (*n* = 70) **Control Group** (*n* = 69) **Setting:** Web based intervention	**Mode: Group** **Study gr:** 12-week access to the ENCOURAGE program with fully automated information **Control gr** = Usual care	**1:** Increased optimism and control over the future (subscale of the constructs empowering outcomes questionnaire) **2:** Distress was measured using the Dutch DT/PL, the European Organization for Research and Treatment of Cancer Quality of Life Questionnaire-Core 30 (QLQ-C30) and the breast cancer specific Quality of Life Questionnaire (QLQ-BR23) **Self-report questionnaire**: six and 12 weeks	No differences between the control and intervention group for primary and secondary outcomes. In clinically distressed patients (*N* = 57), the ENCOURAGE program increased optimism and control over the future at 12 weeks more than in the control group (Cohen’s d = 0.65)
Akturan et al. (2017) [38] Turkey	To investigate the effect of the BATHE therapeutic interview technique on the empowerment of type 2 diabetes mellitus (DM) patients	Cluster RCT **Theory:** not identified	*N* = 112 adult type 2 DM patients. **Study Group**: (*n* = 57) **Control Group**: (*n* = 55) **Setting:** Outpatient primary care	**Mode: Individual** **Study gr:** DM patients of primary care physicians who had taken training in the BATHE therapeutic short interview technique. **Control gr:** patients of physicians without this training	1: A 21-item Turkish version of the diabetes empowerment process scale (DES) was used to assess the participants’ perceptions of empowerment provided by healthcare providers **Self-report questionnaires:** Baseline + in the 6th month	Increase in the DES total score for the intervention group was higher than for the control group (Δ = 10.56 ± 8.97; Δ = 5.64 ± 7.36; *p* < 0.001). The BATHE intervention showed a significant predictor of the DES difference (B: 8.861; CI: 6.092–11.629; *p* < 0.001)
Almeida et al. (2019) [39] Iran	To assess the effectiveness of the Living Harmoniously with Diabetes program in self-efficacy perceptions in patients with type 2 DM	Quasi randomized **Theory:** Health Belief Model (Becker, 1974) [40]	*N* = 42 adult Type 2 DM patients diagnosed for more than a year, and possess skills to manage their disease autonomously **Study Group**: (*n* = 19). **Control Group**: (*n* = 23) **Setting**: Outpatient public health center	**Mode: Group** **Study gr:** Mode: six two-hour group sessions held once a week. Interactive and practical approach, on decision-making and daily problem-solving skills. The educational program was grouped into 4 modules. **Control gr:** Follow the regular surveillance program	1: The Diabetes Empowerment Scale—Short Form (DES-SF) **Self-report questionnaires:** Baseline + 6 weeks	The DES subscale score for “Assessing Dissatisfaction and Readiness to Change” did not change during the study period (*p* = 0.382), whereas “Managing Psychosocial Aspects of Diabetes” and "Setting and Achieving Goals” subscale scores increased significantly
Aslani et al. (2019) [41] Iran	To determine the effect of empowerment on the self-efficacy of Ischemic Heart Disease (IHD) patients admitted to a healthcare center	RCT **Theory:** A Person Centered Model for the Promotion of his/her empowerment (Anderson & Funnell, 2004) [42]	*N* = 56 adult patients with IHD. **Study Gr**: (*n* = 28), **Control Gr**: (*n* = 28) **Setting:** Outpatient at hospital	**Mode: Group** (3–5 pers) **Study gr:** The empowerment program was presented in three 45-min sessions. **Control gr**: Usual care	1: The standard chronic disease self-efficacy scale (CDSES) **Self-report questionnaire:** baseline, after training, 4 weeks, 8 weeks	No significant difference in the mean scores of self-efficacy before the intervention. After the intervention and at the first and second phases of control, the mean score of self-efficacy was higher in the experimental group than in the control group
Chen et al. (2018) [43] China	To examine the effectiveness of patient-centered self-management empowerment intervention (PCSMEI) on self-efficacy, activities of daily living, and rehospitalization of first-time stroke survivors	Two armed single blind Randomized Controlled prospective trial **Theory:** The Health Empowerment Theory (Shearer, 2009) [44]	*N* = 144 adult stroke patients. **Study Group**: (*n* = 72) **Control Group**: (*n* = 72) **Setting:** Hospitalized in-patient and continued post-discharge out-patient	**Mode: Mixed** (**Group + Ind)** **Study gr:** Patient-centered self-management empowerment intervention: 5 daily sessions (day 3–7), one small group setting, discharge instruction + 4 weeks telephone follow up **Control gr:** Conventional nursing (unstructured health education), same number of telephone calls of general social chatting	1: The Stroke Self-Efficacy Questionnaire (SSEQ). 2: The Barthel index (BI) was used to measure performance in ADL **Self-report questionnaire**: baseline, on discharge (T1), 1-month postdischarge(T2) and 3 months postdischarge(T3)	There were significant differences found in the change of self-efficacy between the study group and the control group T1 (β = 3.644; 95% CI [0.728, 6.560]), T2 (β = 4.968; 95% CI [1.322, 8.613]), and T3 (β = 4.252; 95% CI [0.576, 7.928]), reaching statistical levels at T1 (*p* = .014), T2 (*p* = .008) and T3 (*p* = .023)
Cheng et al. (2018) [45] China	To evaluate the effectiveness of a patient-centered, empowerment-based programmed on glycemic control and self-management behavior	Prospective multi-center, single blind, RCT. **Theory:** The Health Empowerment Theory (Shearer, 2009) [44]	*N* = 242 adult patients with poorly controlled Type 2 DM. **Study Group:** (*n* = 121) **Control Group:** (*n* = 121) **Setting** two tertiary hospitals (in-patients)	**Mode: Mixed (Group + Individual)** **Study gr**: Two group sessions (1/ week) + 4 phone based individual sessions on diabetes related empowerment-based self-management **Control gr:** Health education classes and post discharge follow-up	1: Glycemic control (measured by HbA1c) and self-management behaviors **Biometric and survey assessment:** Baseline + 8th- and 20th-week follow-ups	The intervention group exhibited significant improvements in general diet management at the 8th-week (β = 0.740; *p* = 0.013), specific diet management at 8th-week (β = 0.646; *p* = 0.022) and 20th-week (β = 0.517; *p* = 0.043), and blood glucose self-monitoring at both the 8th- (β = 0.793; *p* = 0.009) and 20th-week (β = 0.739; *p* = 0.017) follow-ups
Cheng et al. (2021) [46] China	To report the intervention effects on levels of empowerment, diabetes-specific distress, and quality of life, which are secondary outcomes in the overall study	Randomized, parallel, investigator-blind, controlled trial. **Theory:** The Empowerment Process Model Cattaneo and Champman (2010) [47]	*N* = 242 adult patients with poorly controlled Type 2 DM **Study Group**: (*n* = 121) **Control Group**: (*n* = 121) **Setting** in-patient at 2 hospitals and post discharge telephone follow-up	**Mode: Group** **Study group:** 6 week empowerment based transitional care program (establishing goals, diabetes self-management regimen (dietary management and blood glucose self-monitoring) **Control group:** Two general health education classes and biweekly post-discharge social calls on top of routine care	1: Diabetes Empowerment Scale-Short Form (DES-SF), 2: Diabetes Distress Scale (DDS), and the Audit Diabetes Dependent Quality of Life (ADDQoL) **Self-report questionnaire** baseline, one-week post-intervention, and three-month post-intervention	Participants in the intervention group showed significant improvements on empowerment level [(β = 0.163; 95% confidence interval (CI): 0.011 to 0.316, *p* = 0.036) at one-week post-intervention and (β = 0.176; 95% CI: 0.020 to 0.331, *p* = 0.027) at three-month post-intervention respectively
Cinar et al. (2018) [48] Denmark and TurkeY	To assess the effectiveness of Health Coaching (HC) compared with a health education (HE) intervention on the management of glycemic control and periodontal health	(RCT) (partially randomised patient preference trial (PRPP) design **Theory:** The coaching framework (by the International Coaching Community)	*N* = 302 adults with Type 2 diabetes. **Groups**: First phase: Turkey: *N* = 136 HE: *N* = 101 and HC: *N* = 74 (2010–2012). Second phase: Denmark: *N* = 116 HE: *N* = 78 and HC: *N* = 96 **Setting:** Out-patient medical /dental clinic	**Mode**: **Individual Study group (HC**): 3–4 face-to-face sessions and 2–3 telephone calls. The sessions (20–60 min), with a professional health coach in both countries, focused on maintenance and improvement of lifestyles with the aim of at least a 0.4–0.8% reduction in HbA1c (*P* < 0.05) **Control group (HE):** Received standard lifestyle advice after baseline examination and were invited for two more face-to-face and 1–2 telephone sessions	HbA1c, periodontal treatment need index (CPI), health behaviors and anthropometric measures **Self-report questionnaire** Not incuded The study duration was 12 months (6 months initiation-maintenance, 6 months follow-up)	HbA1c and CPI, were significantly improved in the HC group compared with the HE group (*P* < 0.05) in both countries. No significant change was observed in the other clinical parameters (BMI, LM and BFP) in both groups (*P* ≥ 0.05). In Turkey: The HC group compared with the HE group reported a significantly higher rate of ‘positive change in at least one behaviour’ (85% vs. 60%, *P* = 0.001) No difference in Denmark
Cortez et al. (2017) [49] Brazil	To evaluate the effectiveness of an empowerment program for metabolic control aimed at Brazilian patients in the public health system with type 2 diabetes	Cluster RCT **Theory:** Freirean theories (2007) [50]	*N* = 238 Adults aged 30–80 with type 2 diabetes **Study Group:** (*n* = 127) **Control Group**: (*n* = 111) **Setting:** Ten primary care settings (health units)	**Mode: Mixed (Group + monthly telephone follow-up** between cycles of meetings) **Study gr:** 10 meetings over 12 months. (2 h each). Discussions about diabetes identifying needs and building the foundation for empowerment. **Control gr:** did not attend the educational meetings, but received the same routine care from the health teams	1: A questionnaire that evaluates diabetic knowledge (DKN), user attitudes (ATT) and the self-care questionnaire (ESM). The short form empowerment scale (DES) **Self-report questionnaire** At the beginning of the study, before any educational activity, and at the end of the study **Blood** was collected twice: HbA1c (%), triglycerides (TGL) (mg/dl), total cholesterol (TC) (mg/dl), light density lipoprotein (LDL) (mg/dl), and high density lipoprotein (HDL) (mg/dl)	Participants in SG exhibited a greater reduction in the percentage of HbA1c and DBP, and a greater percentage increase in the scores for selfcare, knowledge and attitudes in comparison to individuals in CG (*p* < 0.05)
Dehghan et al. (2017) [51] Iran	To compare the effect of educating self-empowerment program through the training package and workshop on the life quality of diabetic patients	Pre/post design. **Theory:** Not identified	*N* = 40 adults with type 2 diabetes **Study group:** **(***n* = 20) **Control group: (***n* = 20) **Setting:** An out-patient diabetes clinic	**Mode: Group** **Study gr**: = training workshop, 5 sessions of 1:30 h training in small 5-people groups with methods of improving the daily activities of life, ability to overcome obstacles of the disease and principles of weight control, nutrition, exercise and increase motivation and power of decision- making and management of stress **Control gr**: = training package, a multimedia CD with same content	1: The questionnaire of life quality of diabetic patients **Self-report questionnaire** Baseline + two months after the end of sessions	The research findings showed no significant difference in the scores of life quality between two intended groups before and after intervention (P: 0.570), but a significant difference was found before and after intervention (inter-group) in the scores of life quality in each group (*P* < 0.0001)
Dehghan, et al. (2018) [52] Iran	To investigate the effect of empowerment model-based education on self-efficacy and self-esteem in type 2 diabetic patients	Randomized Control Trial. **Theory:** Discussed theories related to defining self-efficacy (Bandura) etc. but no theory that drove the study	*N* = 90 Adults with type 2 diabetes **Study Group**: *N* = not reported. **Control Group**: *N* = not reported. **Setting**: Out-patient diabetes clinic	**Mode: Group** **Study gr:** 6 sessions of 90-min attendance Empowerment based on the three steps of threat perception, problem- solving and evaluation **Control gr:** Usual care	1: Cooper Smith's adult self-esteem questionnaire and 2: diabetes' self-efficacy. DMSES self-efficacy questionnaire **Self-report questionnaire** Verbal questioning before sessions and six weeks after intervention using self-efficacy and self-esteem questionnaires	There is a significant difference between the two groups in level of self-esteem after the intervention, where the level of self-esteem has increased in the st. group. There was no significant difference between the two groups regarding the level of self-efficacy after the intervention
Doupis et al. (2019) [53] Greece	To investigate the effects of a systematic education program and telephone support on self-reported adherence to oral glucose treatment in patients with type 2 Diabetes	Cluster RCT **Theory:** Not presented	*N* = 457 Adults with type 2 diabetes. **Study Group**: (*n* = 230) **Control Group**: (*n* = 227) **Setting:** Conducted in 45 primary and secondary outpatient diabetes care centers	**Mode: Mixed (Group + telephone)** **Study gr**: standard-of-care with a systematic patient education program (empowerment group) included information on disease knowledge, diet and exercise, use of medications and adherence to treatment and stress + biweekly telephone follow up from doctors **Control gr**: usual care	1: HbA1c, blood glucose, LDL-c; and systolic blood pressure, diastolic blood pressure, proteinuria. 2: Morisky Medication Adherence Scale (MMAS). Health-related quality: the five-level EQ-5D (EQ-5D-5L). The visual analogue scale (EQVAS). Treatment satisfaction: Diabetes Treatment Satisfaction Questionnaire (DTSQ/ DTSQc versions) **Self-report questionnaire** baseline visit and at 4- and at 8-month (± 1 week)	MMAS-4 scores showed that the mean score for the empowerment group was significantly higher compared to control group at 4 months (*p* = 0.023) and 8 months (*p* = 0.043). For the empowerment group, the mean HbA1c was reduced to 7.1% at 4 months (- 0.9% from baseline) and to 7.0% at 8 months (- 1.0% from baseline); for the control group, the respective mean HbA1c levels and reductions from baseline were 7.0% (- 0.7%) and 6.9 (- 0.8%). No sign. between-group differences were observed in other clinical characteristics
Ebrahimi et al. (2016) [54] Iran	To evaluate the effect of empowerment model on indicators of metabolic control in patients with type 2 diabetes	Randomized Control Trial. **Theory:** Cognitive theories by Azobel (1997) [55]	*N* = 113 Adults with Type 2 diabetes **Study Group**: (*n* = 53) **Control Group**: (*n* = 53) **Setting:** an out-patient clinic	**Mode: Group** (five groups of 10) **Study gr** The intervention of empowerment approach training: 5–7 weekly meetings (60–90 min) with two experienced nurses + endocrinologist and a nutritionist, and according to the model’s stages (perception of threat, problem solving, and evaluation) **Control Gr**: Conventional training	HbA1C and laboratory indicators consist of fasting and non-fasting blood sugar, triglyceride, cholesterol, and high- and low-density lipoproteins Measured baseline + 3 months **Self-report questionnaire** Not included	After intervention, there was a significant mean difference in HbA1C (*p* = 0.003), FBS (*p* = 0.004), BS (*p* = 0.003), Cholesterol (*p* = 0.033), Triglyceride (*p* = 0.001) and HDL (*p* = 0.000) in two groups in favour of study gr. LDL (*p* = 0.081) was the only metabolic indicator that showed no significant change after intervention
Fardazar et al. (2018) [56] Iran	To empower patients with type 2 diabetes in order to prevent diabetic foot	Quasi randomized. **Theory**: not presented	*N* = 104 Adult patients with Type 2 diabetes. **Study Gr**: (*n* = 52) **Control Gr**: (*n* = 52) **Setting:** in two diabetes clinics	**Mode: Mixed (Group + ind)** **Study gr:** Foot Care Principles program 4 weekly groups × 40–50 min with empowerment strategies; lectures, practically doing feet examination and special feet exercises, films, practicing, group discussion, question and answer, pamphlets and CDs. + individual counseling about foot care and psychological counseling with a mental health professional and suitable socks for diabetic foot **Control gr:** usual care	An empowerment assessment questionnaire, and foot care behavior checklist **Self-report questionnaire** Baseline, one and 3 months after intervention	The mean score of empowerment and foot care behavior of the experiment group was significantly higher than that of the control group in 1 and 3 months after the intervention (*P* < 0.001)
Hourzad et al., (2018) [57] Iran	to evaluate the effectiveness of an empowering self-management model on the self-efficacy and Sense of Coherence (SOC) in the retired elderly with chronic diseases	Randomized Control Trial. **Theory:** Antonovsky’s theoretical model of salutogenesis (1993)	*N* = 60 with at least one officially diagnosed chronic disease. **Study Gr**: *N* = 30 **Control Gr**: *N* = 30. **Setting:** Out-patient, individual interviews, virtual visits and or telephone	**Mode: Individual Study gr:** A five-stage plan (2 weeks of interviews and in-person trainings followed by 6 weeks implementation 1) self-awareness of changes and expectations; 2) optimal goal setting; 3) planning; 4) adjusting physical, psycho-logical, and social structures; and 5) evaluation. first three stages were performed in two 45-min sessions **Control gr:** routine care = screening tests for common diseases, annual visits by a GP, referral to specialist in case of compli-cations, and a group training on general healthy lifestyle	The Sherer’s self-efficacy and Antonovsky’s SOC questionnaires **Self-report questionnaire** Before and after intervention (after 8 weeks)	The mean self-efficacy scores increased by 9.5±5.32 and 1.7±6.04 in the intervention and control groups, respectively, after the intervention (t=5.20, *P* < 0.001. The mean score of SOC increased by 24.2 ±12.05 and 0.1±13.42 in the intervention and control groups, respectively (t=7.18, *P* < 0.001
Hsiao et al. (2016) [58] Taiwan	To evaluate the impact of participation in empowerment groups on the empowerment and self-care of post-renal-transplant recipients	Randomized Control Trial. **Theory:** discussed theories related to defining concepts but no theory that drove the study? (Orem’s self-care theory)	*N* = 122 patients who had undergone a renal transplant within the past 20 years. **Study Gr**: (*n* = 56) **Control Gr**: (*n* = 66) **Setting:** Out-patient setting	**Mode: Group** **Study gr:** One 2-h meeting every 2 weeks for a total of six meetings. The topics included goal setting, problem solving, coping with daily stress, seeking social support, and staying motivated., **Control gr:** usual care	Empowerment Scale, and Self-Care Scale **Self-report questionnaire:** baseline + 4 weeks after the intervention	The empowerment group reported significant increases both in terms of level of empowerment (F = 5.29, *p* = .023) based on age and time interaction (F = 9.86, p G .001) and in terms of self-care behaviors (F = 7.15, *p* = .009)
Kordshooli et al. (2018) [59] Iran	To investigate the effect of family-centered empowerment model on illness perception in heart failure patients (cognitive and emotional representation)	Randomized Control Trial. **Theory:** Family centered empowerment model by Alhani in 2002	*N* = 70 Patients with heart failure. **Study Gr**: (*n* = 35) **Control Gr:** (*n* = 35) **Setting:** Heart clinic in hospital	**Mode: Group** **Study gr**: Received the family-centered empowerment modeling done in 5 sessions. **Control gr:** usual care	The brief illness perception Questionnaire (BIPQ) **Self-report questionnaire:** baseline + 8 weeks	After the intervention, a significant difference was observed in all of the dimensions of illness perception in favour of the study group, except for time line
Lavesen et al. (2016) [60] Denmark	To explore whether telephone follow-up after discharge may reduce readmission rates, lower mortality, and improve disease management in patients with chronic obstructive pulmonary disease (COPD)	Randomized Control Trial. **Theory:** not identified	*N* = 224 Adult patients with COPD. **Study Gr**: (*n* = 122) **Control Gr**: (*n* = 122) **Setting:** Out-patient nurse led telephone empowerment strategy	**Mode: Individual Study gr:** The nurse-initiated telephone follow- up consisted of two telephone follow-up calls on day 2 and day 30 after discharge. Calls centered on admission, awareness of signs of exacerbations and disease management. **Control gr**: usual care	Quality of Life measured by Rand 36 item short form. 1) The primary outcome was readmission rate. 2) Mortality and disease management were secondary outcomes **Self-report questionnaire:** Questionnaire on day 30 after discharge, Re- admissions and deaths were recorded on day 30 and day 84 after discharge	There was no significant difference in readmission rates or mortality, but significant differences in patients’ assessment of own perception of managing dyspnoea, lung symptoms, ability to react to signs of exacerbation and communicate with health professionals
Lenjawi et al. (2017) [61] QATAR	To assess whether a structured nurse led diabetes educational program is effective in improving glycemic and metabolic parameters among South Asians with type 2 diabetes compared to regular outpatient care	Randomized Control Trial. **Theory:** the theories of the health belief model, change in locus of control, and patient empowerment	*N* = 460 Adults with type 2 diabetes.. **The Study Gr:** (*n* = 230) **Control Gr** (*n* = 230) **Setting:** Community setting	**Mode: Group** **Study gr:** A theory based nurse-led, diabetes educational program that is 8 h long, divided into four sessions each lasting 2 h, and held once weekly **Control gr:** Usual care	The primary outcome was the improvement in HbA1c and other metabolic parameters, including lipid profile, albumin/creatinine ratio, blood pressure, and body mass index **Self-report questionnaire:** Not included	The intervention group had statistically significant improvements in HbA1c (-0.55%, *p* = 0.012), fasting blood sugar (-16.6 mg/dl, *p* = 0.022), albumin/ creatinine ratio (-3.09, p \ 0.001), and HDL cholesterol (?6.08 mg/dl, *p* < 0.0001), compared to the controls. There was no sta- tistically significant difference between the groups in systolic blood pressure or diastolic blood pressure, nor BMI
Li et al. (2020) [19] China	To assess the effects of a motivational interviewing (MI) based patient empowerment program (PEP) on Type 2 Diabetes patient self-management compared to traditional diabetes health education	Randomized Control Trial. **Theory:** Motivational Interviewing by Miller/Rollnick: strongly rooted in the person-centered approach of Carl Rogers (1951, 1959, 1980)	*N* = 225 Adult Type 2 DM **The Study Gr** (*n* = 117) **The Control Gr:** (*n* = 108) **Setting:** Out- patient setting	**Mode: Group** **Study gr:** received a four-session PEP in small groups over 1 month by trained nurses and doctors. **Control gr:** received the traditional lecture style health education on diabetes	Problem Areas in Diabetes (PAID), patient enablement index (PEI), mental health, patient satisfaction **Self-report questionnaire:** baseline, post-activity and 3 months	The PEP has a significant effect on improving diabetes-related distress, but MI was not significantly different from the traditional health education programs when it comes to the readiness to change
Macedo et al. (2017) [62] Brazil	The objective of the present study was to evaluate the adherence and empowerment shown by people that engaged in these group activities	Randomized Control Trial. **Theory:** A person-centered model for the promotion of his/her empowerment (Anderson & Funnell, 2005) [42]	*N* = 183 Adult patients with Type 2 diabetes. **The Study Gr**: *N* = 72 **The Control Gr:** *N* = 111. **Setting:** Out- patient clinic	**Mode: Group** **Study gr:** seven group meetings, of two hours with five steps to encourage to think about their condition (based on the Behavior Change Protocol.) topics: 1) problem definition; 2) identification and handling of feelings; 3) definition of goals; 4) elaboration of a care plan; 5) evaluation **Control gr:** Usual care	The validated instruments of adherence to self-care practices for diabetes mellitus (ESM) and empowerment for self-care in diabetes mellitus, short version (Diabetes Empowerment Scale-Short Form – DES-SF), and assessment of HbA1c **Self-report questionnaire:** Baseline + at three-month	A statistically significant decrease (< 0.001) in the value of glycated hemoglobin and an increase in the scores of adherence to self-care and empowerment scales were found for participants in the intervention group (< 0.001)
Maryam et al. (2017) [63] Iran	To evaluate the effect of empowerment program based on telenursing in caregivers of patients on functional capacity and dyspnea in elderly patients with heart failure (HF)	Randomized Control Trial and prospective study **Theory:** A person-centered model for the promotion of empowerment (Anderson & Funnell, 2005) [42]	*N* = 75 Patients with HF. **Study Gr. #1**: *N* = 25; **Study Gr.#2:** *N* = 25 **Control Gr**: *N* = 25 **Setting:** In-person training sessions, 3-months telephone follow-up call	**Mode: Individual** **Study gr**: 1 and group 2. The Patients with caregivers received education by a nurse. During three months, only caregivers of patients in group 2 were followed via telephone and received advice on physical activity, diet, medication, and vital signs monitoring of the patients. **Control gr**: Usual care	Six Minute Walking Test and dyspnea was measured via Burg Scale Baseline + 3 months **Self-report questionnaire:** Not included	After the 3-months intervention there were no significant differences between the three groups (*p* = 0.14)
Moein et al. (2017) [64] Iran	To investigate the effect of a self- empowerment program on self-efficacy in patients with type 2 diabetes	Randomized Control Trial **Theory:** A person-centered model for the promotion of his/her empowerment (Anderson & Funnell, 2005) [42]	*N* = 50 Adult Type 2 diabetics. **Study Gr**: (*n* = 25) **Control Gr**: (*n* = 25) S**etting**: a diabetes center	**Mode: Group** **Study gr**: five steps program conducted in eight 45-min sessions two days a week for four consecutive weeks **Control gr**: usual care	Diabetes self-efficacy questionnaires **Self-report questionnaire:** Baseline + two months after the intervention	At the end of the study, a significant difference was observed between the mean self-efficacy scores in the two groups (55.71 ± 13.25 in the intervention group vs. 40.24 ± 17.55 in the control group, *P* = 0.001)
Musavinasab et al. (2016) [65] Iran	To determine the effect of a self-management empowerment model on the Sense of Coherence (SOC) among elderly patients with cardiovascular disease	Quasi randomized controlled trial. **Theory:** Antosovsky’s theoretical model of salutogenesis	*N* = 96, patients with cardio-vascular disease. **Study Gr** (*n* = 48) **Control Gr** (*n* = 48) (received educational booklet). **Setting:** In-patient	**Mode: Group** **Study gr:** 4 steps: 1) instill self-awareness of changes in physical psychological and social capacities, 2) optimum goal setting. 3) planning, using the goals based on solutions proposed by the elderly and the areas of self-management in the empowerment model. The first three stages were performed in two 45-min sessions. **Control gr:** usual care	Antonovsky’s standard SOC Scale, which were completed by face- to-face interviews with the elderly **Self-report questionnaire:** Baseline + after intervention	A significant difference between the mean total score and the dimensions of SOC in the experimental group and the control group (*p* = .0001): the SOC scores of the experimental group were increased after the intervention
Naik et al. (2019) [21] United States	To evaluate the effectiveness of Healthy Outcomes Through Patient Empowerment (HOPE) (proactive population screening plus telephone delivery of a collaborative goal setting intervention) compared with enhanced usual care (EUC)	Randomized Control Trial. **Theory**: not identified	*N* = 225 US veterans with depression and diabetes. **Study Gr:** (*n* = 136). **Control Gr:** (*n* = 89) **Setting**: in both in-patient and 6 affiliated community-based outpatient clinics	**Mode: Individual** **Study gr:** Intervention Healthy Outcomes Through Patient Empowerment (HOPE) included 9 telephone sessions with 24 trained health care professionals using collaborative goal-setting and behavioral activation methods **Control gr:** EUC and notification of high-risk status. + related educational materials	The Patient Health Questionnaire–9 (PHQ-9) telephone screening for depression and HbA1c, **Self-report questionnaire** baseline, 6 months, and 12 months	Mixed results; a significantly higher proportion of intervention participants achieved and maintained clinically significant responses of depression symptoms at 12 months, but did not find such improvements for glycemic levels at 12 months
Ramli et al. (2016) [66] Malaysia	To evaluate the effectiveness of the EMPOWER-PAR intervention (multifaceted chronic disease management strategies designed based on the Chronic Care Model (CCM0) in improving clinical outcomes for patients with TYPE 2 diabetes using existing health care resources in the Malaysian public primary care setting	Randomized Control Trial. **Theory**: EMPOWER-PAR intervention (based on The Chronic Care Model) Wagner, E., 1998 [67]	*N* = 888 Patients with Type 2 diabetes. **Study Gr**: (*n* = 471) **Control Gr**: (*n* = 417) **Setting**: 10 public primary care clinics	**Mode: Individual** **Study gr:** During the 1-year intervention period, all patients in the intervention arm were required to be seen at least twice by the Chronic Disease Management (CDM) team from each clinic. The EMPOWER-PAR intervention was designed based on the six interrelated elements of the CCM **Control gr**: Five clinics continued with usual care	Primary outcome: change in the proportion of patients achieving glycemic target of HbA1c < 6.5% (48 mmol/mol). Secondary outcomes were measured by changes in the proportions of patients achieving the following targets: BP ≤ 130/80 mmHg; BMI < 23 kg/m2; Waist Circumference (WC) < 90 cm for men, < 80 cm for women; Total cholesterol (TC) ≤ 4.5 mmol/L; Triglycerides (TG) ≤ 1.7 mmol/L; Low density lipoprotein cholesterol (LDL-c) ≤ 2.6 mmol/L; and High density lipoprotein cholesterol (HDL-c) ≥ 1.1 mmol/L **Measures:** baseline and at 1-year follow-up. **Self-report questionnaire**: Not included	The intervention group showed significant reduction in the mean HbA1c compared to control, which showed an increase in the mean HbA1c (intervention: − 0.1%, SE ± 0.06 vs. control: 0.2% SE ± 0.09, *P* = 0.003). For diastolic BP, although both groups showed an increment at 1-year follow-up, the intervention group had a significantly lower mean change in diastolic BP compared to the control group (intervention: 0.4 mmHg, SE ± 0.43 vs. control: 1.9 mmHg SE ± 0.47, *P* = 0.02)
dos Santos et al. (2017) [68] Brazil	To compare the adherence and empowerment of patients with type 2 diabetes mellitus for self-care practices and glycemic control in group education strategies and home visits	Cluster Randomized Controlled Trial. **Theory**: not identified	*N* = 238 Patients with type 2 diabetes **Study Gr:** *N* = 93 **Control Gr:** *N* = 111 **Setting**: A clinical setting and home visits	**Mode: Mixed (group education and home visits)** **Study gr:** Empowerment intervention with 10 meetings (120 min) and 8 home meetings (90 min). Educational strategies focused on adherence and empowerment for self-care using the behavior change protocol **Control gr:** usual education, 2 telephone calls and 2 semiannual meetings	HbA1c and Brazilian version of the Diabetes Empowerment Scale-Short Form (DES-SF); Self-Care Questionnaire in Diabetes Mellitus (ESM); and HbA1c **Self-report questionnaire + blood test:** baseline + 1 year follow up	Both educational strategies contributed to the improvement of adherence and empowerment for self-care. However, group education when compared individually with the control group and the home visit was the strategy that presented the best result in glycated hemoglobin
Shin et al. (2016) [69] Korea	To examine the effects of the Empowerment Program for Self-management (EPSM) on stroke patients’ self- efficacy, self-management behavior, and functional recovery	Randomized Control Trial. **Theory:** Freire Theories (The Educational Theory): Freire, 2007 [50]	*N* = 77 Stroke patients **Study Gr:** *N* = 41 **Control Gr**: *N* = 36. **Setting**: An ambulatory rehabilitation center of a subacute hospital	**Mode: Mixed (Group + individual)** **Study gr**: The final 12-month EPSM consisted of three parts: (i) 12-week lifestyle modification group sessions (60–90 min) (i.e. exercise, education on low-sodium recipes, smoking cessation and drinking), and discussions about goal setting and problem solving; (ii) individual phone calls by research nurses (iii) monthly maintenance follow-up meetings C**ontrol gr:** conventional including aerobics or yoga and counselling about medication adherence	Self-efficacy, social support and self-care behaviors. BP control and renal function were measured as clinical outcomes **Self-report questionnaire + clinical tests** baseline, 6 months and 12 months	Significant interactions of group by time for self-efficacy (*P* < 0.001) and self-care behaviour (*P* = 0.019). Blood pressure control at 12 months showed a significant improvement in the empowered group compared with the control group (82.8% versus 56.8%, *P* < 0.014). systolic blood pressure (*P* = 0.006) and renal function (*P* < 0.001), showed significant interactions of group by time
Souza et al. (2017) [70] Brazil	To evaluate the effect of home visits on the adherence and empowerment of users with type 2 diabetes for self-care practices	Randomized Control Trial. **Theory:** Discussed theories related to defining concepts etc. but no theory that drove the study	*N* = 145 Patients with Type 2 diabetes. **Study Gr:** (*n* = 34) **Control Gr:** (*n* = 111) **Setting**: out-patient home visits	**Mode: Individual (**Home visits + monthly telephone) **Study gr:** home visits (each 2 h = 14 h totally in 3 cycles, 3 month intervals between) based on the Behavior Change Protocol in diabetes. = 31 questions in five steps: (1) problem identification; (2) identification and approach of feelings; (3) goal setting; (4) the care plan; and (5) assessment and experience about the care plan **Control gr:** conventional follow-up + three telephone calls from the research nurses and pamphlets about diabetes	The Diabetes Empowerment Scale-Short Form (DES-SF) and self-care measurements with diabetes (ESM) **Self-report questionnaire: baseline:** baseline, and intragroup between before and after the study period	In the comparison between intervention and control groups, the effect on the diabetes self-care (ΔESM) in the intervention group was considered statistically different from the control group (***p* < 0.001**). Regarding empowerment (DES-SF), there was a statistically significant in- crease of the median score in both groups (*p* < 0.05). However, this increase was not considered statistically different between the two groups (*p* = 0.607)
Sit et al. (2016) [71] Hong Kong	To examine the effects of the empowerment intervention (HEISS)on stroke patients’ self- efficacy, self-management behavior, and functional recovery	Randomized Control Trial. **Theory:** Health Empowerment Theory by Shearer, (2009) [44]	*N* = 210 patients with stroke. **Study Gr**: (*n* = 105) **Control Gr:** (*n* = 105) **Setting:** an ambulatory rehabilitation center of a subacute hospital	**Mode: Mixed: (group + telephone follow up)** **Study gr:** 13-week empowerment intervention part 1 had 6-weekly small group sessions from week 3 to week 8; groups worked with nurse facilitator for stroke self-management to begin personal goal setting and action planning. Part 2: home-based during weeks 9–13 with biweekly telephone follow-up calls **Control gr**: receiving usual ambulatory rehabilitation care	Self-efficacy and self-management behavior were assessed using the Chinese Self-Management Behavior Questionnaire **Self-report questionnaire: baseline:** baseline (T0), 1 week (T1), 3 months (T2), and 6 months (T3) postintervention	SG reported better self-efficacy in illness management 3-month (*P* = 0.011) and 6-month (*P* = 0.012) post -intervention, better self-management behaviors at all follow-up time points (all P,0.05), apart from medication adherence (P.0.05). SG had significantly better functional recovery (Barthel, all P,0.05; Lawton, all P,0.001), compared to CG
Tabari et al. (2018) [72] Iran	To examine the effect of education based on family-centered empowerment model on the quality of life of elderlies with chronic obstructive pulmonary disease (COPD)	Non-randomized clinical trial. **Theory**: Family centered empowerment model by Alhani in 2002	*N* = 80 Older adults with COPD. **Study Gr**: (*n* = 40) **Control Gr**: (*n* = 40) **Setting**: in a pulmonary clinic	**Mode: Group** **Study gr**: 4 stages of education based on family-centered empowerment (threat perception, knowledge translation and improvement, problem solving, **Control gr**: usual care	Quality of Life Questionnaire (SF-36) **Self-report questionnaire:** Baseline + 3 months	After the intervention, the difference between the mean score of quality of life in the two groups was statistically significant (*P* < 0.001)
Theeranut et al. 2018 [73] Thailand	To evaluate the short-term effects of the empowerment program on the short-term effects of the empowerment program on HbA1c and lipid profiles in an inpatient setting	Quasi-experimental intervention study **Theory:** Not identified	*N* = 57 Patients with type 2 diabetes. **Study Gr:** (*n* = 27) **Control Gr:** (*n* = 30) **Setting:** An in-patient setting	**Mode: Individual Study gr:** received the empowerment course three times prior to discharge. Consisting of 4 steps 1. building patient self-awareness, 2. implementing nursing interventions to empower patients, 3. evaluating outcomes and 4. monitoring and supporting pat empowerment **Control gr**: standard care	Body weight (BMI), HbA1c, HDL-c, and LDL-c Baseline, three and six months **Self-report questionnaire:** Not included	The mean HbA1c and LDL-c levels of the intervention group were significantly lower than those of the control group at three and six months (*p* < 0.05), The mean HDL-c level of the intervention group was significantly higher than the control group six months (1.54 vs 1.29 mmol/L; *p* value < 0.001). The average BMI of the intervention group was significantly lower at six months (22.74 vs 25.54 kg/m2; *p*-value = 0.016)
Üzar-Özetin et al. (2019) [74] Turkey	To assess the feasibility of an integrated empowerment program for cancer survivors, to examine the effect of the program on the resilience and Post Traumatic Growth (PTG) levels of cancer survivors, and relationships between resilience and PTG at the end of the program and in the follow-up	Randomized Control Trial. **Theory:** Post traumatic Growth (PTG) Theory (Tedeschi RG, Calhoun LG)	*N* = 89 Cancer patients.. The Study Group: (*n* = 45). **Control Gr**: (*n* = 44) **Setting:** an outpatient oncology unit	**Mode: Group Study gr:** Received a structured 10-session empowerment program with different content (cancer experience; communication and self-expression; assertiveness skills; self-perception and roles; coping skills) **Control gr:** Usual care	Post traumatic Growth Inventory (PTGI) and the Resilience Scale for Adults (RSA) **Self-report questionnaire:** baseline, end of intervention + one month after the intervention	Posttraumatic growth and resilience levels significantly improved in the intervention group compared with the control group both at the end of and a month after the program
Vahedian-Azimi et al. (2016) [75] Iran	To determine if a hybrid cardiac rehabilitation (CR) program using the Family-rehabilitation (CR) program Centered Empowerment Model (FCEM) with standard CR will improve patient quality of life, perceived stress and state anxiety of patients with myocardial infarction (MI)	Randomized Control Trial. **Theory:** Family centered empowerment model by Alhani in 2002	*N* = 70 Patients status post myocardial infarction. **Study Gr:** (*n* = 359) **Control Gr**: (*n* = 35) **Setting**: a coronary care unit	Mode: **Mixed** (telephone with nurse + 21 support group webinars) **Study gr:** intervention of a hybrid cardiac rehabilitation program using the Family- Centered Empowerment Model (FCEM) in four stages: (1) determining perceived threat; (2) self-efficacy; (3) improving self-esteem and (4) process and outcome evaluations **Control gr:** standard cardiac rehabilitation	Family-Centered Empowerment Model (FCEM), the 36-Item Short Form Health Survey (SF-36), the perceived stress, and State and Trait Anxiety questionnaires **Self-report questionnaire: baseline:** The HRQoL dimensions, perceived stress and anxiety were assessed at baseline and at 3 months postintervention. Empowerment was measured at baseline and at 10 days postintervention	The quality of life results in the FCEM group showed significant improvement compared with control ( *p* < 0.0001). Similarly, the perceived stress and state anxiety results showed significant improvement compared with control (*p* < 0.0001). No significant difference was found either within or between groups for trait anxiety
van Puffelen et al. (2019) [76] Netherlands	To improve type 2 diabetes patient self-management and quality of life in the first years of living with the disease	Randomized Control Trial. Theory: not identified	*N* = 168 Patients with type 2 diabetes **Study gr:** (*n* = 82) **Control Gr:** (*n* = 86) **Setting:** in an out-patient setting	**Mode: Group** **Study gr**: self- management support program, consisting of three monthly 2-h interactive sessions and one booster session three months after the last session **Control gr**: received (a single educational lecture) with their partners	Self-care was assessed with the revised Summary of Diabetes Self-Care Activities measure (SDSCA) **Self-report questionnaire:** baseline, 2 + 8 months from baseline	The intervention group showed a significantly higher increase in physical activity and fruit and vegetable intake immediately after the program, whereas the low baseline levels of diabetes distress remained unaffected
Visser et al. (2018) [77] Netherlands	To test the effect of a blended care intervention Group-Medical Consultations MY-GMC (and online app and online SGS) to an individual BC follow-up visit (care as usual)	Randomized Control Trial (. **Theory**: not identified	*N* = 109 Breast cancer patients. **Study Gr:** (*n* = 59 = **Control Gr**: (*n* = 50) **Setting**: one academic and two general hospitals in the Netherlands	**Mode: Mixed (Group + online follow up)** **Study gr:** participated in a face-to-face GMC combined with a tablet-based online app, consisting of three online support group sessions (SGS) and additional information). **Control gr**: One individual outpatient follow-up visit	**1:** The Symptom checklist -90 (SCL-90), The Dutch Diabetes Empowerment Scale (Dutch DES-20) **2:** The 8-item Cancer Worry Scale (CWS), Quality of life was measured by the EORTC-QLQ- C30 and the EORTC-BR23 **Self-report questionnaire:** baseline, 1 week, 3 and 6 months after the visit	No between-group differences were found for the primary outcomes distress and empower- ment. More themes were discussed in GMCs compared to individual visits. Significantly more patients experienced peer-support in GMCs
Young et al. (2020) [78] United States	To evaluate the effectiveness of a nurse coaching program using motivational interviewing (MI) paired with mobile health (mHealth) technology on diabetes self-efficacy and self-management for persons with type 2 diabetes	Randomized Control Trial. **Theory:** They discussed theories related to defining self-effect etc. but no theory that drove the study	*N* = 287 Persons with type 2 diabetes **Study Gr**: (*n* = 132). **Control Gr:** (*n* = 155) **Setting:** in-person orientation with the nurse coach, followed by telephone sessions	**Mode: Individual + telephone follow up** **Study gr:** 6 individual sessions using a counseling style based on the concepts of MI. Sessions promoted mutual goal setting, enhanced self-efficacy in health behavior change, and assist to derive meaning from data to reinforce choices and behaviors. In-person orientation with the nurse coach, then telephone sessions every 2 weeks for 3 months (6 contacts total) **Control gr**: usual care	1. Diabetes Empowerment Scale (DES-SF) 2. Depression severity (Patient Health Questionnaire-9 (PHQ-9) **Self-report questionnaire:** Web-based surveys at baseline, 3 months (coinciding with the end of the intervention or 3 months from baseline), and 9 months	The participants in the intervention group had significant improvements in diabetes self-efficacy (DES -SF), 0.34; 95% CI –0.15,0.53; *P* < .01) and a decrease in depressive symptoms compared with usual care at 3 months PHQ-9; 0.89; 95% CI 0.01–1.77; *P* = .05), with no differences in the other outcomes. The differences in self-efficacy and depression scores between the 2 arms at 9 months were not sustained
Zamanzadeh et al. (2017) [79] Iran	To investigate the effect of distance education by telephone and short message service on empowering patients with type 2 diabetes who were referred to the Urmia Diabetes Association	Randomized Control Trial. **Theory**: not identified	*N* = 66 Patients with type 2 diabetes.. **Study Gr:** (*n* = 33) Control Gr: (*n* = 33) **Setting:** at the Urmia diabetes association in Iran	**Mode: Individual** **Study gr**: received an educational text message daily and instructive phone calls three days a week for three months along with usual care. **Control gr:** usual care	Diabetes Empowerment Scale (DES) **Self-report questionnaire:** Baseline + after intervention	The empowerment of the intervention group compared with the control group significantly improved after three months of distance education (*p* < 0.00, EF = 1. 16)
Zoun et al. (2019) [80] Netherlands	To study the effectiveness of the SemCAD or Self- Management for Chronic Anxiety and Depression) ZemCAD on quality of life, symptom severity and empowerment compared to usual care	Multicenter RCT **Theory**: not identified	*N* = 141. **Study Gr:** zemCAD: (*n* = 70) **Control Gr:** CAU: (*n* = 71) **Setting:** 12 specialized outpatient mental health care services in the Netherlands	**Mode: Individual** **Study gr:** ZemCAD consists of three parts.; 13 sessions over 26 weeks. First, educationd about the nature of their chronic disorder, (coping). 1: 3 weeks/ weekly sessions, individual treatment plan, identify symptoms… 2: coaching and treatment phase of 14 weeks with sessions every second week. (social skills/ problem solving) 3. 9 weeks with sessions every three weeks (action plan, deal with crisis). **Control gr:** Usual care (outpatient mental health care)	Quality of life was measured with the World Health Organization Quality of Life instrument, Brief version (WHOQOL-BREF); the Beck Anxiety Inventory (BAI); the Patient Health Questionnaire-9 (PHQ-9); Empowerment is assessed using the Netherlands Empowerment List (NEL) **Self-report questionnaire** Baseline, 6, 12, and 18 months after baseline	Results at 18-month follow-up regarding to quality of life and symptom severity, showed no significant differences between the ZemCAD group and the CAU group, except on the ‘social relationships’-domain (d = 0.37). With regard to empowerment a significant difference between both groups was observed in the total empowerment score and one empowerment dimension (d = 0.45 and d = 0.39, respectively)

### Synthesis

Meta-analyses were performed using RevMan V5.2 software. To summarize continuous data, the pooled mean difference (MD) and 95% confidence interval (CI) were calculated. Given the included interventions were delivered in different modes, formats, sessions and duration, random-effect models were used in the pooled analysis [81]. The I^2^ metric describes the percentage of total variation across studies due to heterogeneity. The Q-value was used to examine the degree of heterogeneity.

## Results

**Fig. 1:**
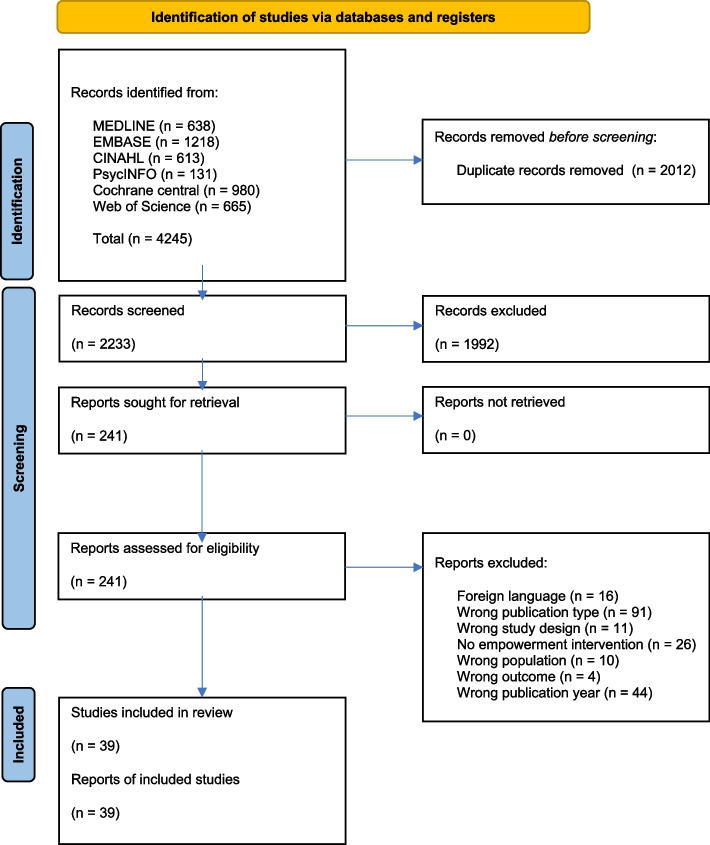
PRISMA flow chart. The figure details our search and selection process applied during the systematic review

This systematic review yielded 39 empowerment-focused intervention studies conducted among 8,011 participants. We retrieved 2,233 reports after the removal of duplicates and excluded 1,992 reports based on the title, abstract, and keywords, leaving 241 reports that were assessed for eligibility by reviewing the full-text (Fig. 1).

### Characteristics of the studies included

The characteristics of the individual studies are summarized in Table 2. Of 39 included studies, 32 (85%) were RCTs [19, 21, 37, 38, 41, 43, 45, 46, 49, 52–54, 57–64, 66, 68–71, 74–80]. Seven studies used other methods including five quasi-randomized trials, [39, 56, 65, 72, 73] one pre-post design, [51] and one partially randomized patient preference trial [48]. All studies were published in English within the 5-year period spanning 2016–2020, inclusive.

A total of 15 different countries were represented in the review. Most studies were conducted in Iran (*n* = 13), followed by Brazil (*n* = 4), China (*n* = 4), Netherlands (*n* = 3), United States (*n* = 2) and Turkey (*n* = 2). One study was conducted in each of Denmark, Denmark/Turkey, Greece, Hong Kong, Malaysia, Portugal, Qatar, South Korea, Taiwan, and Thailand (see Table 2).

The studies included a variety of chronic diseases with most conducted among patients with a diagnosis of Type 2 diabetes mellitus (*n* = 22) followed by cardiovascular disease (*n* = 3), stroke (*n* = 3), chronic obstructive pulmonary disease (COPD) (*n* = 2), heart failure (*n* = 2) cancer (*n* = 3). One study included patients who had undergone a renal transplant, one was conducted among veterans with depression and diabetes, one study included patients suffering from chronic anxiety and depression. In addition, one study included patients with at least one officially diagnosed chronic disease. Mean age ranged between 46.9 (SD 5.5) [52] and 73.29 years (SD 8.6) [69].

The types of health care professionals (HCP) involved in intervention delivery varied to a large degree across studies: 22/39 studies included nurses, 8/39 studies used multidisciplinary teams, 3/39 studies used physicians, and one study each with dentists, research psychologists, and a research team. Four studies were unclear about the types of HCP who were involved; [46, 51, 64, 79].

Retention rate of the intervention groups ranged between 38 and 100%. Retention rate of the control groups ranged between 46 and 100%.

### Intervention characteristics

Intervention settings, modalities and content varied across studies. Settings included outpatient or community clinics (*n* = 32), inpatient settings (*n* = 1) and mixed settings (*n* = 5) where patients began the intervention as inpatient and continued post-discharge in the outpatient setting. One study did not identify an intervention setting [77].

Twelve interventions were individual based, of which ten were reported as successful [21, 38, 48, 57, 63, 66, 70, 73, 78, 79] while two were unsuccessful [60, 80]. Seventeen interventions were group based, of which fourteen were successful [19, 41, 46, 52, 54, 58, 59, 61, 62, 64, 65, 72, 74, 76]. Ten interventions combined more than one method of delivery (e.g., group and individual), of which nine were successful [43, 45, 46, 49, 53, 56, 69, 71, 75]. The number of sessions ranged from 3—22, with large variations in frequency. Web-based interventions ranged in duration from 3—22 weeks.

A variety of educational approaches were used across interventions including lectures, counseling, informational booklets, tests, workshop discussions, interactive methods, motivational strategies and social support strategies. Multiple approaches were combined in many interventions performed face-to-face, digitally, or by telephone. A variety of resources were used to support intervention delivery including diary logs, reflective journaling, computer tablets, and developed learning modules. Details of health care professional (HCP) training were not generally reported.

In terms of content, 26 interventions reported a theory-based foundation. In total, 13 theoretical frameworks were used across studies in support of intervention content, revealing a broad conceptual understanding of empowerment. The most recurrent underlying theories were patient empowerment, presented in 10 studies [43, 45, 46, 48, 49, 59, 62, 64, 71, 72, 75]. Twelve studies did not use any theoretical framework, and four studies referred to theories to define concepts only without providing further evidence of use [52, 58, 70, 78]. The most common theory, the Person-Centered Model for the Promotion of his/her Empowerment [42] was used by four studies [41, 62–64]. Three studies [43, 45, 71] used the Health Empowerment Theory [44] and three studies [59, 72, 75] used the Family-Centered Empowerment Model [82]. Two studies [49, 69] used Educational Theory [50] and two studies [57, 65] used the Theory of Salutogenesis [83]. In addition, Almeida, Correira de Sousa, et al. [39] and Lenjawi, Mohamed, et al. [61] used the Health Belief Model [40]. Other theories such as the theory of problem solving [37] and the coaching framework [48] were represented by single studies.

Intervention content varied depending on study aims and assessed outcomes. Twelve studies aimed to understand the effectiveness of empowerment strategies on self-efficacy, self management behaviors, or readiness to make behavioral changes [19, 41, 43, 52, 56–59, 63–65, 71]. For example, Sit, Chair, et al. [71] evaluated a 13-week empowerment intervention on self-management behavior, self-efficacy and functional recovery, delivered across six weekly nurse facilitated group sessions and four weeks of telephone follow-up.

Ten studies aimed to increase empowerment of patients, using patient-reported outcome measures (PROMS) [38, 39, 46, 49, 62, 70, 75, 77–79]. For example, Vahedian-Azimi, Miller, et al. [75] described a cardiac rehabilitation program using the Family-Centered Empowerment Model delivered through 21 support group webinars to improve physical and mental health of post myocardial infarction (MI) patients [75].

Four studies described empowerment interventions that were aimed at improving quality of life [37, 51, 72, 80].

Eleven studies used empowerment interventions to improve glycated hemoglobin (HbA1c) along with other metabolic measures and patient-reported outcome measures (PROMS) [21, 43, 48, 49, 53, 54, 61, 62, 66, 68, 73].

There were large variations in study duration, follow-up time and measurement points. Most studies had a relatively short follow-up (2–12 weeks), but ten studies (26%) collected data after 6–9 months of follow-up.

Only five studies (12.8%) measured effects 1–2 years post-intervention [66, 68, 69, 75, 80].

### WHO empowerment components

We assessed intervention content in relation the WHO conceptual framework of empowerment [15] by applying the four fundamental components and assessing whether they were incorporated within intervention design: (1) patient participation and understanding of their role; (2) patient acquisition of enough knowledge so they can engage with their health care provider; (3) patient skills; and (4) the creation of a facilitating environment [15] (Table 3). Patient skills (35/39) and patient participation (29/39) were addressed in most studies. Seven studies clearly described patient knowledge that enabled better engagement with HCP. All studies incorporated at least one component and 13 studies targeted three components. No studies addressed all four components. Components reflecting knowledge, making patients able to engage with the health care provider and the facilitating environment were scarcely reported.

**Table 3:** Overview of empowerment components in the included studies according to the WHO definition of empowerment

	Study reference	**Component 1** Understanding by the patient of his/her role	**Component 2** Acquisition by patients of sufficient knowledge to be able to engage with their healthcare provider	**Component 3** Patient’s skills	**Component 4** Facilitating environment / Support by others	Comments to the assessments	Number of WHO components used
1.	Admiraal et al. 2017 [37]	X		X		1: Problem orientation and identification 3: Problem solving (increased coping)	2
2.	Akturan et al. 2017 [38]				X	4: Therapeutic interview techniques by doctor	1
3.	Almeida et al. 2019 [39]	X		X		1: Modul 2: How diabetes affects my daily life 3: Decision making and daily problem-solving skills	2
4.	Aslani et al. 2019 [41]		X?			2: Educational booklet (disease, symptoms, lifestyle and medication)	1
5.	Chen et al. 2018 [43]	X		X	X	1: Promoting knowledge and self-management 3: Self-care skills, enhancing self-efficacy 4: facilitate a collaborative relationship (nurse, family caregiver)	3
6.	Cheng et al. 2018 [45]	X		X		1: Self-management knowledge 3: Self-management skills and self-efficacy, goalsetting, autonomy	2
7.	Cheng et al. 2021 [46]	X		X		1: Self-management knowledge 3: Self-management skills and self-efficacy, goalsetting, autonomy **Same intervention as Cheng 2018 **[45]	2
8.	Cinar et al. 2018 [48]	X		X		1: Educational materials (a physical exercise DVD and chi-balls, cookery books for DM2 patients, oral hygiene brochures 3: healthy lifestyle, self-efficacy, diabetes coping skills	2
9.	Cortez et al. 2017 [49]	X	X	X		1: Explore the problem, identify and discuss feelings and meanings 2: Produce effective interactions between health professionals and users 3: Set goals, create a care plan	3
10.	Dehghan et al. 2017 [51]	X		X		1: improving the daily activities of life, principles and the objectives of weight control, nutrition, exercise 3: Strategies of decision- making, increasing motivation & stress management	2
11.	Dehghan et al. 2018 [52]	X		X		1: The perception of patients increased by knowing the nature and complications of late diabetes 3: problem-solving, promotion of self-efficacy through skills such as blood glucose monitoring, foot care, physical activity, maintenance, insulin and dietary intake	2
12.	Doupis et al. 2019 [53]	X		X		1: Material on disease knowledge, diet and exercise, use of medications and adherence to treatment 3: Attainment of treatment goals included diet, physical activity, adherence to prescribed medication etc	2
13.	Fardazar et al. 2018 [56]	X		X		1: General introduction of diabetes, its mechanism, and complications 3: Foot care principles (daily foot examinations, nail care and foot care, appropriate footwear, stress management	2
14.	Ebrahimi et al. 2016 [54]	X		X		1: Education was provided in four areas including diet, exercise, medication, and foot care 3 They discussed their problems and possible solutions;, practical skills were trained	2
15.	Hourzad et al. 2018 [57]	X	X	X	-	1; Self-awareness of changes and understanding their personal level of performance and expectations 2. Empowering them to receive timely information from the medical team on various aspects of their disease 3: Optimal goal setting; adjusting physical, psychological, and social structures	3
16.	Hsiao et al. 2016 [58]	X	-	X	X	1: identification of problem areas for self-care behaviors after renal transplant 3: setting goals, solving problems, coping with renal transplant and daily stresses 4: seeking social support	3
17.	Lavesen et al. 2016 [60]	X	-	X	-	1: Supporting active participation in own disease management 3: Symptom management, inhalation medicine, medicine	2
18.	Lenjawi et al. 2017 [61]	X	-	X	-	1: Taking the main responsibility for managing their disease, behavioral modifications, and psychological adjustment 3: Goal-setting skills, and coping skills, taking blood glucose, target comorbid conditions such as hypertension, dyslipidemia, and microalbuminuria	2
19.	Li et al. 2020 [19]	-	X	-	X	2: Knowing Diabetes 3: Motivation on Diabetes Self-Care, Healthy Diet and Physical Exercise	2
20.	Macedo et al. 2017 [62]	X	-	X	-	1: Make decisions consciously and independently about their health care 3: Develop care plan and meet goals which allowed adherence and empowerment to execute self-care practices	2
21.	Maryam et al. 2017 [63]			X	X	3: Education: disease, medications, diet, lifestyle, evaluating peripheral edema, stress management, behavior change, self-management and follow-up of treatment 4: Telephone support to caregivers about the same	2
22.	Moein et al. 2017 [64]	X		X	X	1. Understanding diabetes and related complications 3. Using glucometer for measuring blood glucose level; where to store insulin; how to use insulin syringes; how to do insulin injections, etc.) 4: Using peer support motivation and leaning	3
23.	Musavinasab et al. 2016 [65]	X		x		1: Instill self-awareness of changes and expectations of themselves 3. Goal setting, empowerment strategies to identify and adopt adaptation and self-management	2
24.	Naik et al. 2019 [21]		X	X	X	2. Advocate for their health through active communication with their clinicians 3.Action plans, improve wellness, diet, physical activity, medication management, and relaxation 4. The coach-patient relationship	3
25.	Ramli et al. 2016 [66]		X	X	X	2. Improve provider-patient communication, 3. Decision Support, 4. have a productive interaction between the empowered CDM team and the informed, empowered T2DM patients, Self- Management Support	3
26.	Kordshooli et al. 2018 [59]	X		X		1. The nature of disease, pathophysiology, etiology and clinical demonstrations 3. Increasing the self- efficacy, self-esteem and self- control	2
27.	dos Santos et al. 2017 [68]	X	-	X	-	1..Exploration of the problem and feelings and emotions 3. Lify style change: feeding frequency and fiber intake; 4) nutrients, reading of food labels and physical activity	2
28.	Shin et al. 2016 [69]	-	-	X	-	3.. Exercise, low-sodium recipes, smoking cessation and healthy drinking, and group discussions about goal setting and problem solving	1
29.	Sit et al. 2016 [71]	-	-	X	-	3.personal goal setting and action planning. Self-efficacy activities to develop self-management skills and articulating participants’ health needs	1
30.	Souza et al. 2017 [70]	X	-	X	X	1. Identification and approach of feelings 3. Problem solving, goal setting, self-care practice, diet 4 Support of interactive dynamics through dialogues with nurses	3
31.	Tabari et al. 2018 [72]	X	-	X	X	1.improved his/her perceived sensitivity in relation to the disease and its control 3. Problem solving, increase self-efficacy, self-esteem and self-control 4. booklets and pre-prepared educational pamphlets for the participants and active family members	3
32.	Theeranut et al. 2018 [73]	X	-	X	-	1. Self-reflection technique to identify problems related to diabetes care 3. self-care, decision making, goal setting, and practice of the patients diet control, exercise, stress and coping	2
33.	Young et al. 2020 [78]	-	-	X	-	3. Mutual goal setting, enhance self-efficacy in health behavior change, using Motivational interviewing	1
34.	Zamanzadeh et al. 2017 [79]	?	?	X	-	3: Education message daily by SMS and necessary education given over the phone three times a week	1
35.	Zoun et al. 2019 [80]	X	-	X	X	1: Individual treatment plan, identify symptoms and daily activities, keep a log of symptoms, 3: coping with the chronic disorder 4: An action plan to re-establish social contacts and improve daily living activities	3
36.	Üzar-Özetin et al. 2019 [74]	X	-	X	X	1: Cancer experience and effects 3: Coping styles, stress management, self- expression, say NO 4: Resources for social support	3
37.	Vahedian-Azimi et al. 2016 [75]	X	-	X	X	1: Awareness and cognition addressed, rehabilitation plan + insight in illness severity 3: Problem solving 4: Follow up with support group webinars on relevant topics	3
38.	van Puffelen et al. [76] 2019	X	-	X	X	1: Discussing maladaptive illness perceptions 3: Basic information about diabetes, creating goals, create stepwise actionplans 4: Exploring and discussing (un)helpful ways of support	3
39.	Visser et al. 2018 [77]	X	x	-	-	1: Support groups on survivorship 2: Discussing psychosocial themes related to Breast cancer survivorship to improve consultations with MD	2
Total number of studies		29	7	35	14		

### Outcomes and instruments

Empowerment was presented as the primary outcome in 11/39 studies [37–39, 56, 58–60, 70, 76, 78, 79]. Clinical outcomes were presented as a primary outcome in 12 studies. In several studies, empowerment was used as a secondary outcome; in the remaining studies primary and secondary outcomes were not defined (Table 4).

**Table 4:** Overview of measurements used in the included studies of empowerment intervention

Measures	Number of studies (n)	Study citations
**Patient-reported outcome measures (PROMS)**		
**Empowerment measures**		
Diabetes Empowerment scale (short form) (DES-SF)	7	Akturan et al., 2017 [38]; Almeida et al., 2019 [39]; Young et al., 2020 [78], Cheng et al. 2018 [45], Macedo et al., 2017 [62], Souza et al. 2017 [70], dos Santos et al. 2017 [68]
Diabetes Empowerment Scale (DES)	2	Zamanzadeh et al., 2017 [79], Visser et al., 2018 [77] (Dutch DES-20),
Empowerment Scale	1	Hsiao et al., 2016 [58]
the Family Centered Empowerment Model (FCEM) questions	1	Vahedian-Azimi et al., 2016 [75]
Netherlands Empowerment List (NEL)	1	Zoun et al. 2019 [80]
Construct Empowering Scale Outcomes (CEO)	1	Admiraal et al. 2017 [37]
Empowerment Questionnaire	1	Fardazar et al. 2018 [56]
Total number of measures / studies	7/ 14	
**Self-Management / Self-care measures**		
Self-care Questionnaire (EMS)	4	Macedo et al., 2017 [62], Souza et al. 2017 [70], Cortez et al. 2017 [49], Hsiao et al. 2016 [58],
Empowerment Program for Self-management (EPSM)	1	Shin et al. 2016 [69]
the Chinese Self-Management Behavior Questionnaire	1	Sit et al., 2016 [71]
Self-Management Behavior	1	Cheng et al., 2018 [45]
the Summary of Diabetes Self-Care Activities Measure (SDSCA)	1	van Puffelen et al. 2019 [76]
Total number of measures / studies	5 / 8	
**Quality of life measures**		
Diabetes Quality of Life (QOL) measures	3	Dehghan et al., 2017 [51], Lavesen et al., 2016 [60]; Tabari et al., 2018 [72]
Audit Diabetes Dependent Quality of Life (ADDQoL)	1	Cheng et al. 2018 [45]
European Organization for Research and Treatment of Cancer Quality of Life Questionnaire-Core 30 (QLQ-C30)	1	Admiraal et al., 2017 [37]
Breast cancer specific QOL (QLQ-BR-23)	1	Admiraal et al. 2017 [37]
the World Health Organization Quality of Life Brief Version (WHOQOL-BREF)	1	Zoun et al. 2019 [80]
Health Related Quality of Life EQ-5D (EQ-5D-5L),	1	Doupis et al., 2019 [53]
EORTC-QLQ- C30 and the EORTC-BR23	1	Visser et al. 2018 [77]
Total number of measures / studies	7 / 9	
**Self-efficacy measures**		
the Diabetes Self Efficacy Scale (DMSES)	2	Dehghan et al. 2018 [52], dos Santos et al., 2017 [68]
The Standard Chronic Disease Self-Efficacy Scale (CDSES)	1	Aslani et al. 2019 [41]
Stroke Self-Efficacy Questionnaire (SSEQ)	1	Chen, Chen, Xiangyu, et al. 2018 [43]
Cooper Smith’s Adult Self-esteem questionnaire	1	Dehghan et al. 2018 [52]
the Sherer’s Self-efficacy Scale	1	Hourzad et al.,2018 [57]
Diabetes Self-efficacy Questionnaires	1	Moein et al. 2017 [64]
Total number of measures / studies	6 / 7	
**Distress/Anxiety measures**		
Dutch Distress Scale (DDS)	1	Admiraal et al. 2017 [37]
Beck Anxiety Inventory (BAI)	1	Zoun et al. 2019 [80]
Diabetes Distress Scale (DDS)	1	Cheng et al. 2018 [45]
The perceived stress Questionnaire	1	Vahedian-Azimi et al. 2016 [75]
State and Trait Anxiety questionnaires	1	Vahedian-Azimi et al. 2016 [75]
Cancer Worry Scale (CWS)	1	Visser et al. 2018 [77]
Patient Health Questionnaire-9 (PhQ-9)	3	Naik et al. 2019 [21], Zoun et al. 2019 [80], Young, 2020 [78]
Total number of measures / studies	7 / 9	
**Health issues/Treatment related measures**		
The Barthel Index (BI), (Functional assessment)	1	Chen, Chen, Xiangyu, et al. 2018 [43]
the Problem Areas in Diabetes (PAID)	1	Li et al. 2020 [19]
User Attitudes (ATT)	1	Cortez et al. 2017 [49]
Diabetes Treatment Satisfaction Questionnaire (DTSQ / DTSQc)	1	Doupis et al. 2019 [53]
Foot Care Behavior Checklist	1	Fardazar et al. 2018 [56]
the Periodontal Treatment Need Index (CPI)	1	Cinar et al. 2018 [48]
36-Item Short Form Health Survey (SF-36)	1	Vahedian-Azimi et al. 2016 [75]
Total number of measures / studies	7 / 7	
**Illness perception**		
Brief Illness Perception Questionnaire (BIPQ)	1	Rahimi Kordshooli et al., 2018 [59]
the Resilience Scale for Adults	1	Üzar-Özetin et al., 2019 [74]
Antonovsky Sence of Coherence (SOC) Questionnaires	2	Hourzad et al. 2018 [57], Musavinasab et al. 2016 [65]
The Symptom checklist -90 (SCL-90)	1	Visser et al. 2018 [77]
Total number of measures / studies	4 / 5	
**Other PROMS**		
Diabetes Knowledge,Questionnaire (DKN)	1	Cortez et al. 2017 [49]
Post Traumatic Growth Inventory	1	Üzar-Özetin et al., 2019 [74]
Morinsky Medication Adherence Scale (MMAS),	1	Doupis et al. 2019 [53]
the Visual Analogue Scale (EQVAS)	1	Doupis et al. 2019 [53]
Total number of measures / studies	4 / 4	
**Total PROMS**	46	
**Biochemical measures**		
HbA1C	11	Macedo et al., 2017 [62], Cortez et al. 2017 [49], Chen et al., 2018 [43], Cinar et al. 2018 [48], Doupis et al. 2019 [53], Ebrahimi et al. 2016 [54], Lenjawi et al. 2017 [61], Naik et al. 2019 [21], Ramli et al. 2016 [66], dos Santos et al. 2017 [68], Theeranut et al. 2018 [73]
Fasting and non-fasting Blood Sugars	1	Ebrahimi et al. 2016 [54]
Total Cholesterol / Cholesterol (HDL-c and LDL-c)	4	Doupis et al. 2019 [53], Ebrahimi et al. 2016 [54], Ramli et al. 2016 [66], Theeranut et al., 2018 [73]
Triglycerides	2	Ebrahimi et al. 2016 [54], Ramli et al. 2016 [66]
High and low density lipoproteins	3	Cortez et al. 2017 [49], Ebrahimi et al. 2016 [54], Ramli et al. 2016 [66]
Lipid profile/albumin/creatinine ratio	1	Lenjawi et al. 2017 [61]
Total number of measures / studies	6 / 22	
**Anthropometric measurements**		
Waist Circumference	1	Ramli et al. 2016 [66]
Body Mass Index	3	Lenjawi et al. 2017 [61], Ramli et al. 2016 [66], Theeranut et al., 2018 [73]
Blood Pressure	3	Lenjawi et al. 2017 [61], Ramli et al. 2016 [66], Doupis et al. 2019 [53]
Total number of measurements / studies	3/7	
**Functional testing**		
Six Minute Walking Test	1	Maryam et al. 2017 [63]
the Burg Test	1	Maryam et al. 2017 [63]
Total number of functional tests / studies	2/2	

The diverse array of outcome measures included: empowerment, self-management, sense of coherence, illness perception, anxiety and depression, self-efficacy, QOL, knowledge, self-care management, medication adherence, diabetic foot prevention, patient enablement, and post-traumatic growth. Clinical outcomes included: HbA1C; total cholesterol; triglycerides; high- and low-density lipoproteins; serum creatinine; and fasting/non-fasting blood sugars. Anthropometric measurements included waist circumference, body mass index, ejection fraction, and blood pressure.

The most common measurement instruments used included: the Diabetes Empowerment Scale-Short Form (DES-SF) alone or in combination with other instruments (*n* = 7) and the Diabetes Quality of Life (QOL) measures (*n* = 3) (Table 4).

Many studies combined several Patient Reported Outcome Measures (PROMS) or used these in combination with clinical measures. A total of seven specific empowerment-focused PROMS were used in 14 different studies. Different variations of the Diabetes Empowerment Scale were used in nine studies. Eight studies used self-management or self-care PROMS.

### Intervention effects

**Fig. 2:**
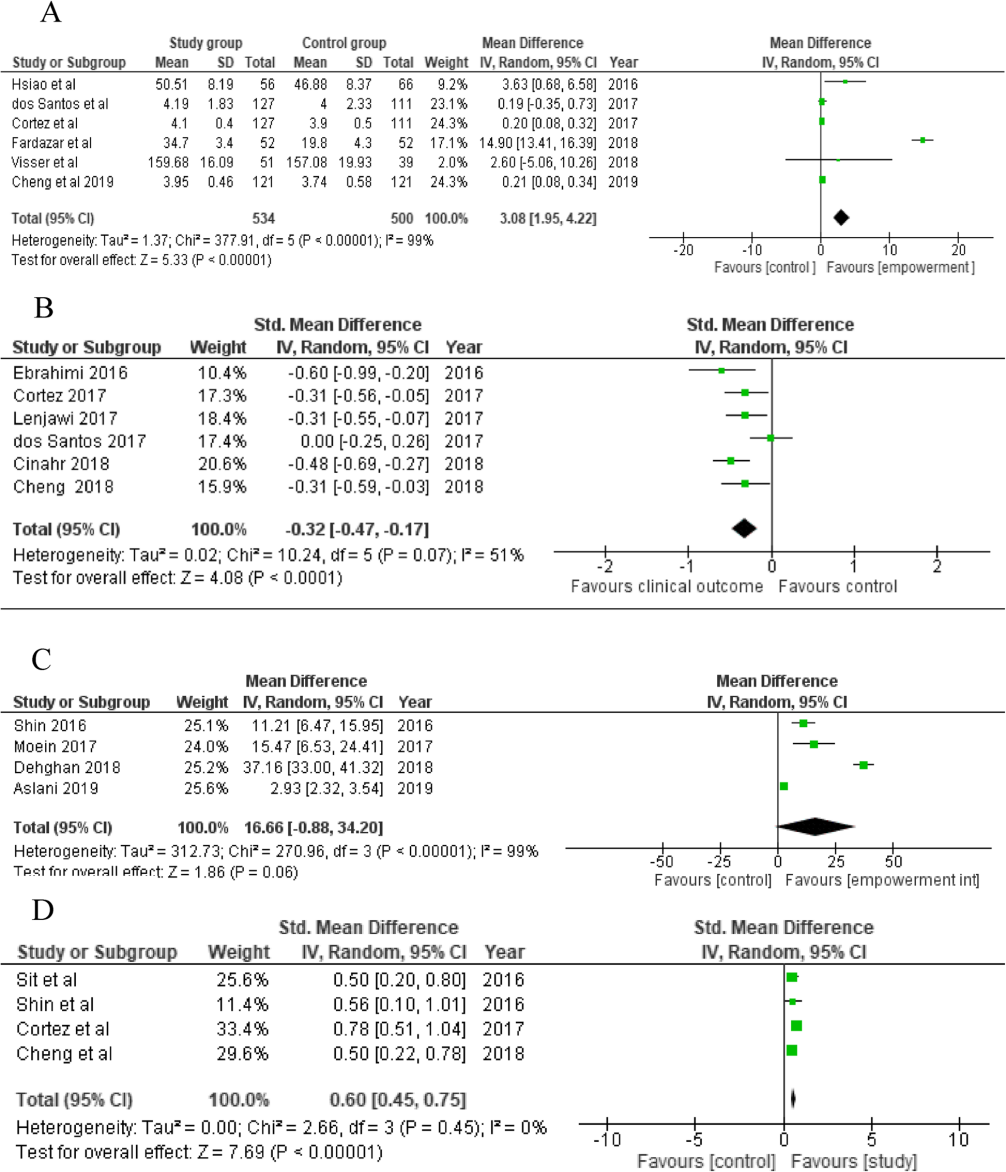
Forest plot of the meta-analysis of group-format empowerment interventions versus control using the Empowerment Scale (2A), HbA1c (2B), self-efficacy (2C) and Self-Management / self-care (2D)

Few studies were assessed as similar enough to be included in meta-analysis. Six studies reported group-format interventions using variants of the Empowerment Scale [46, 58, 77]; three of which also included an additional individual follow up, [49, 56, 68] with a total of *n* = 1,034 patients. Pooled results showed strong evidence for an effect favoring interventions, however with high heterogeneity (SMD 3.08; 95% CI, 1.95 to 4.22, *p* < 0.0001; I^2^ = 99%, *p* < 0.00001) (Fig. 2A). In six other studies of group-format interventions representing *n* = 1,434 participants, [45, 48, 49, 54, 61, 68] the pooled result showed strong evidence for a reduction in HbA1c (MD, − 0.32; 95% CI, − 0.47 to − 0.17; *p* < 0.0001; moderate heterogeneity I^2^ = 51%, *p* = 0.07) (Fig. 2B). Four other studies of group-format interventions [41, 52, 64, 69] also showed strong evidence of a pooled effect on self-efficacy (MD 1.86 95% CI, 0.81 to 3.24), *p* = 0.001; however, with high heterogeneity I^2^ = 99%, *p* < 0.06 (Fig. 2C). Four studies showed evidence of a positive pooled effect on self-management / self-care (MD 7.69), *p* =  < 0.001, I^2^ = 0% (Fig. 2D) [45, 49, 69, 71].

**Fig. 3:**
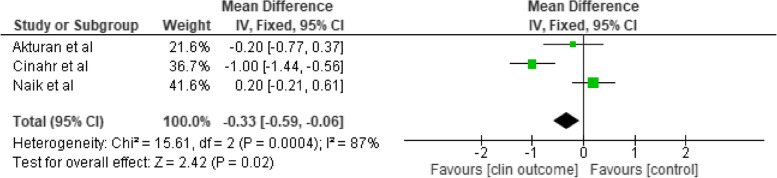
Forest plot of the meta-analysis of individual-format empowerment interventions versus control using HbA1c (E)

Three individual-format interventions measuring HbA1c found statistically significant improvement (MD, -0.33; 95% CI, − 0.59 to – 0.06; *p* = 0.02; (with high heterogeneity I^2^ = 87%, *p* = 0.0004), (Fig. 3) [21, 38, 48].

Of all studies, 32/39 reported strong evidence for changes on the primary outcome of interest in favor of the intervention group. Of these, 10/32 were conducted in individual format; 15/32 were conducted in group format; and 6/32 in mixed formats.

One-third (13/39) of studies that included an empowerment measure found significant improvement in empowerment scores [38, 39, 46, 49, 56, 58, 62, 68, 70, 77–80]. Six studies also found improvement post-intervention in self-care management measures.

Of 12 studies that measured self-care management behavior, self-efficacy or readiness to make behavioral changes, effects were found in eight studies for example empowerment and foot care behavior, [56] empowerment and self-care behaviors, [58] and self-efficacy [43, 57]. Of ten studies that aimed to improve patient empowerment, eight were focused on type 2 diabetes, and all reported improvement in empowerment and self-care management [38, 39, 46, 49, 62, 70, 78, 79]. The four studies describing empowerment interventions aimed at improving QOL, optimism and control over life found mixed results, for example Tabari, Razi SH, et al. [72] reported an improvement in QOL among elderly people with COPD.

### Quality appraisal

**Fig. 4:**
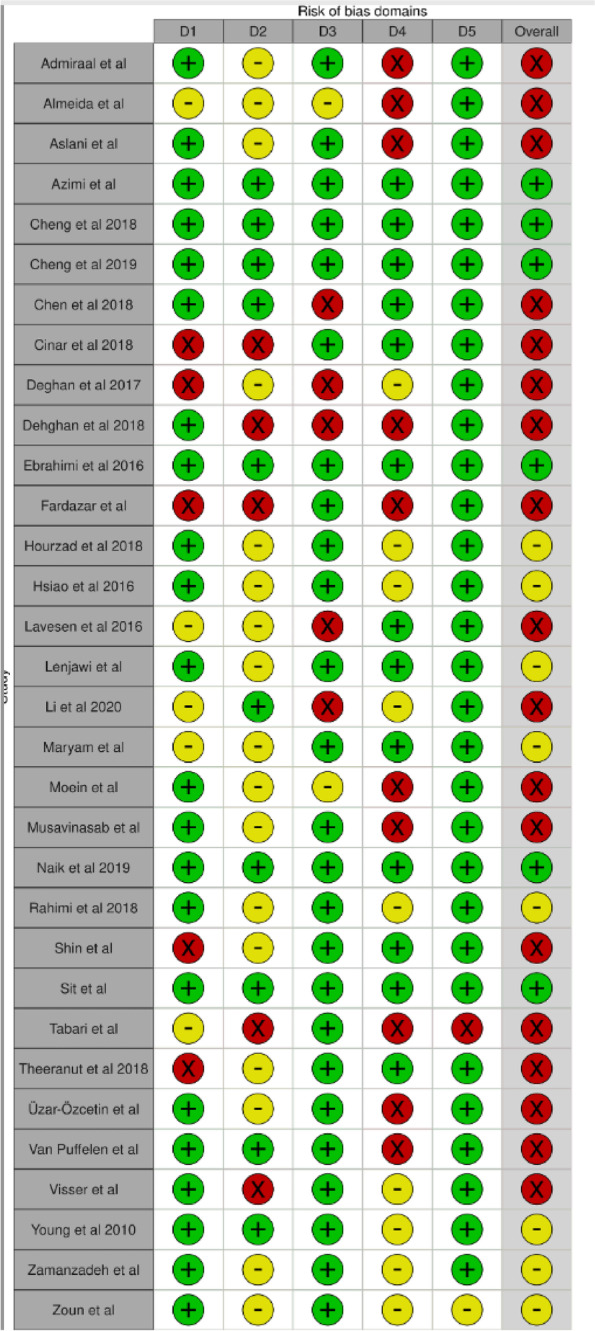
Risk of Bias Domains for individual randomized studies using Rob2 tool. Domains: D1: bias arising from the randomization process, D2: bias due to deviations from intended intervention, D3: bias due to missing outcome data, D4: bias in the measurement of the outcome, and D5: bias in the selection of the reported result. Legend: Red (x) = high risk of bias; Yellow (-) = unknown risk of bias; Green ( +) = low risk of bias

**Fig. 5:**
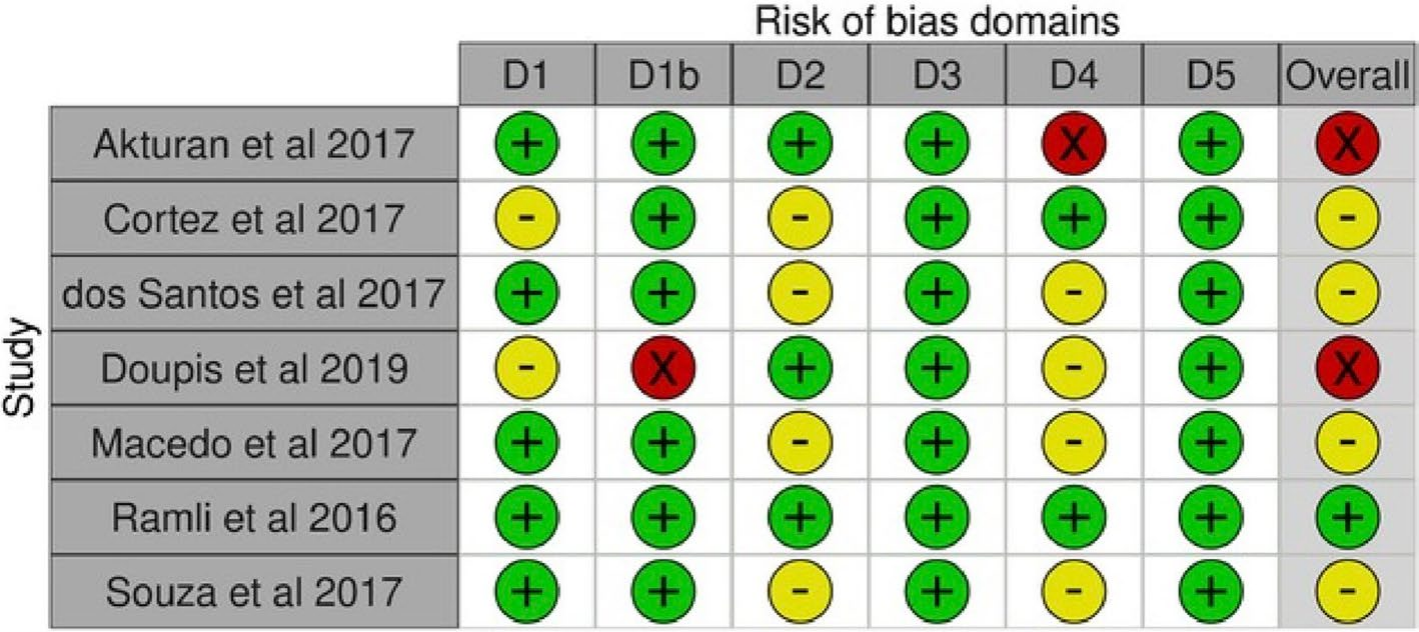
Risk of Bias Domains for cluster randomized studies using Rob2 tool. Domains: D1: bias arising from the randomization process, D1b: Bias arising from the timing of identification and recruitment of individual participants in relation to the timing of randomization. D2: bias due to deviations from the intended intervention, D3: bias due to missing outcome data, D4: bias in the measurement of the outcome, and D5: bias in the selection of the reported result. Red (x) = high risk of bias; Yellow (-) = unknown risk of bias; Green ( +) = low risk of bias

All studies were assessed using the Risk of Bias 2 (ROB2) tool, with 19% of the individual randomized studies evaluated as having an overall low risk of bias [84]. More than half of these studies (56%) showed high risk of bias, and a quarter had some concerns. In studies with a overall high risk of bias, concerns arose primarily from the randomization process and/or possible deviation from the intended intervention. However, in terms of selective reporting of results, more than three-quarters of studies (30/39) received a low ROB score (Figs. 4 and 5).

Among the seven cluster randomized studies, only one was evaluated as having a overall low risk of bias [66]. All studies scored low risk in bias due to missing outcome and bias in selection of the reported results. The most problematic domains in these studies were bias due to deviations from the intended intervention and bias in measurement of the outcome. Four studies were evaluated as having some concerns in overall bias and two studies had a high risk of bias overall (see Supplementary file 2 (Table 5) for reasons for the selected assessments and Supplementary file 3 for a Graph Summary plot (ROB2).

No studies were excluded from the review or effect presentations due to poor methodological quality.

## Discussion

This review of empowerment interventions covering thirteen diagnostic categories expands on prior findings regarding content, measures and efficacy of empowerment approaches in chronic disease.

We found that a majority (58%) of studies used a theory or framework, a finding that contrasted with Werbrouk et al. [3] who detected a much larger proportion of studies (81%) that employed a theory-based intervention. These findings suggest that incorporation of theory in intervention design has declined in recent years. We also reviewed intervention setting, modality and content to clarify consistency with WHO’s empowerment definition. Overall, we found little consistency in how empowerment was deployed conceptually in the design of interventions and we did not identify any studies that included all four of WHO’s fundamental constructs for empowerment. Most studies (85%) employed just two components. However, WHO describes these components as fundamental, signifying that each is equally important for establishing efficacious interventions. This approach is supported in assessments of the concept within the literature that consider empowerment as a dynamic process that addresses the sense of powerlessness and loss of control that is common among individuals’ who are a managing a chronic disease [5]. Aujoulat et al. [85] described empowerment as consisting of an inter-personal dimension (a process of communication and education in which knowledge, values and power are shared in provider-patient interactions) and an intrapersonal dimension (patients’ process of personal transformation). Dialogue between health care providers and patients, co-creation of knowledge, a patient-centered approach, enhancement of patient competencies, and active participation have also been identified as antecedents of patient empowerment while self-management and improved quality of life have been identified as potential outcomes of the empowerment process [11, 86]. The WHO definition is generic and not specific enough on the above mentioned aspects of the empowerment process, however, the first fundamental component includes the concepts of patient participation, patient knowledge and patient skills, and therefore, it reflects a person-centered perspective to a certain degree. Based on our study and use of the WHO, we recommend the development of an updated and unified definition of empowerment that capture the importance of the person-centered perspective and emphasize the dialogue with health care professionals in order for empowerment to happen. There is a need for a more thorough analysis of the personal transformation which develops the individual`s ability to cope and the transfer of power between health care professionals and patients. Furthermore, Health coaching has developed as an important approach to promote self-awareness and empowerment in patients with chronic disease [87–89] and is an interesting field for future studies.

Notably, nurses were responsible for delivering the intervention in half (56%) of studies. In addition, some studies described interprofessional collaboration in intervention delivery where nurses often were team-members. These findings are consistent with the philosophy of patient-centered care, which implies patient activation and patient participation in practice [90]. According to WHO [15], patient participation is the first of the fundamental components of empowerment. In order to utilize patients’ inherent resources for health, tools and interventions to exploit these under-utilized resources are needed [10]. Patient participation and patient activation can therefore be seen as complementary strategies for achieving patient-centered care, which in turn can affect patient empowerment [11]. Most studies were implemented in outpatient or community-based settings where community and public health nurses often have the responsibility for intervention delivery and follow-up of persons with chronic disease. Primary care is a highly relevant setting for the development and delivery of empowerment-focused strategies and interventions by interprofessional teams and by nurses independently.

In descriptive analyses, we found that most interventions delivered in group-format (13/17) and individual-format (10/12) were reported as successful. Of ten interventions that combined more than one method of delivery (e.g., group and individual), nine were successful. These findings diverged from the meta-analysis undertaken by Werbrouk et al. [3] of 23 empowerment interventions among patients with somatic chronic diseases that found an effect in favor of individual format interventions on empowerment-based PROMS. In contrast, our results suggested that empowerment interventions were more effective when conducted in groups or in combinations of group and individual formats. In our meta-analyses of six studies in group-formats measuring HbA1c, we also found strong evidence in favor of empowerment interventions, which did not hold in individual formats. These findings were consistent with a meta-analyses of 21 studies that compared group-based diabetes self-management education with routine treatment, a waiting list control and no intervention, finding strong evidence for an effect on HbA1c at 6, 12, and 24 months of follow-up [91]. The superiority of the group-directed format of empowerment interventions was also confirmed in Chen et al.’s review [23] that found improvements in blood pressure and reductions in cholesterol among people with diabetes.

Our review also found that empowerment interventions were effective when measuring several clinical markers and PROMS, including empowerment, self-efficacy and self-care management. These findings aligns with results reported by Chen and I-Chuan [23] suggesting that empowerment interventions improved the health status, psychological status and quality of life among patients with chronic disease. These findings also confirm that development of broadly applicable empowerment interventions may be a promising approach for future intervention development focused on improving self-care management and health among patients with chronic disease.

We summarized outcome measures used to evaluate empowerment, self-care management and clinical outcomes and found inconsistencies in measurement. According to WHO, empowerment is a unique concept with the potential to influence patient activation and self-management [15]. However, we found that only 36% of studies used any empowerment scale, 31% used clinical outcome measures, 21% measured self-management, but only 5% of studies used all three measures. These findings reveal a gap in knowledge on the essential role of empowerment in pathways that include patient activation, self-care management, and clinical outcomes.

Recent disease specific reviews had been conducted among patients with hypertension [92], chronic metabolic diseases [26] and people with type 2 diabetes mellitus [91]. Our review contributes to highlighting the importance of empowerment interventions among patients with chronic disease in general. We assessed the overall quality of the evidence and found that only one in five studies had a low risk of bias while the majority were high risk. Other recent reviews found similar levels of quality. In their review of internet-based intervention studies, Kuo et al. [26], found that fewer than one-third (29%) of the 21 reviewed studies reported allocation concealment, blinding of outcome assessments, or role of study personnel. Overall, our review revealed inconsistencies concerning definition of patient empowerment consistent with Mora et al.’s [12] descriptive review. Using the WHO [15] definition of the empowerment process and ensuring that all four fundamental components of empowerment are covered in intervention design, may provide more consistency in future research and clinical practice. Future studies on patient empowerment should also consider including both an empowerment measurement tool as well as measures of self-management and clinical outcomes to assess the effect of empowerment strategies.

### Strengths and limitations

Strengths of this review included the wide range of settings and populations included and consistent outcome measures that enabled meta-analyses on individual and group format of the interventions as well as on some PROMS. To our knowledge this is the first review of empowerment interventions including such a diversity of chronic diseases. Limitations included the possibility that we could have missed reports not indexed within the six databases searched, from references cited within our included studies, and in grey literature. Another limitation is that eHealth studies has not been included if they did not include empowerment in the title or abstract. There are many important concepts related to methods and approaches in empowerment interventions that could have been used in the search, i.e. patient participation, patient activation, patient engagement, shared decision making, health coaching and more. Our choices may have had the consequences that we have lost some studies that otherwise might have added to the findings. The sample size of most studies included in our meta-analyses was small. The intervention effect on the PROM measures should be interpreted with caution due few studies eligible for inclusion and high heterogeneity of modes, operational definition of empowerment and measurement tools. Furthermore, it was challenging to extract and categorize interventions because of considerable variability in intervention design.

## Conclusion

In conclusion, our findings demonstrate that empowerment interventions in chronic disease contains essential components that contribute to strengthening patients’ capability for self-care management and health in chronic disease and are important in order to attain WHO`s sustainable development goals. Future studies investigating the role of empowerment in chronic disease should consolidate conceptual understandings by using WHO’s empowerment components and investigate the role of empowerment in pathways that include patient activation, self-care management, and clinical outcomes. Group-format or mixed format interventions delivered in outpatient or community health settings and Primary Care are especially suitable to facilitate patients’ process of taking control of their health. 

### Supplementary Information

**Additional file 1: Supplementary file 1.** Literature Search Strategies from Ovid Medline, Embase, Cinahl, APA PsycInfo, Cochrane Central and Web of Science.

**Additional file 2: Supplementary file 2. Table 5.** Risk of Bias (ROB) assessments and short reasons for the selected assessments.

**Additional file 3: Supplementary file 3.** Graph Summary plot ROB2.

## Data Availability

Dataset are available through the corresponding author upon reasonable request.

## Abbreviations

CI: Confidence Interval
COPD: Chronic Obstructive Pulmonary Disease
DES-SF: Diabetes Empowerment Short Form
HbA1c: Glycated Hemoglobin
HCP: Health Care Professionals
MD: Mean Difference
MI: Myocardial Infarction
PICO: Population/Patient/Problem, Intervention, Comparison, and Outcome
PRISMA: Preferred Reporting Items for Systematic Reviews and Meta-Analyses
PROMS: Patient Related Outcome Measures
PROSPERO: International Prospective Register of Systematic Reviews
QOL: Quality of Life
RCT: Randomized Control trial
ROB2: Risk of Bias 2
SD: Standard Deviation
SMD: Standard Mean Difference
WHO: World Health Organization
