# Supplementary material for: Empowerment interventions designed for persons living with chronic disease - a systematic review and meta-analysis of the components and efficacy of format on patient-reported outcomes
Source: BMC Health Serv Res. 2023 Aug 25;23:911. doi: 10.1186/s12913-023-09895-6 (PMC10463815; doi:10.1186/s12913-023-09895-6)

**Supplementary file 1:** Literature Search Strategies from Ovid Medline, Embase, Cinahl, APA PsycInfo, Cochrane Central and Web of Science

| Database(s): **Ovid MEDLINE(R) and Epub Ahead of Print, In-Process & Other Non-Indexed Citations and Daily**1946 to March 24, 2020 | |
| --- | --- |
| Search Strategy: |  |
| **#** | **Searches** |
| 1 | exp Power, Psychological/ |
| 2 | empower*.tw,kf. |
| 3 | 1 or 2 |
| 4 | Cancer Survivors/ or (Survivors/ and (exp Neoplasms/ or cancer.tw,kf.)) or ((cancer or neoplasm*) and surviv*).tw,kf. |
| 5 | (Fatigue/ or fatigue*.tw,kf.) and (exp Neoplasms/ or (cancer or neoplasm*).tw,kf.) |
| 6 | 4 or 5 |
| 7 | exp Lung Diseases, Obstructive/ |
| 8 | (chronic or "long-term" or "long term" or longterm or lifelong or "life long" or "life-long" or permanent or persisting).tw,kf. |
| 9 | 7 and 8 |
| 10 | exp Pulmonary Disease, Chronic Obstructive/ |
| 11 | exp Asthma/ |
| 12 | (((chronic or "long-term" or "long term" or longterm or lifelong or "life long" or "life-long" or permanent or persisting) adj3 (pulmonary or lung*1 or airway* or airflow* or bronch* or respirat*)) or emphysema*).tw,kf. |
| 13 | (COPD or COAD or COBD or AECB).tw,kf. |
| 14 | (asthma*1 or asthmatic).tw,kf. |
| 15 | 9 or 10 or 11 or 12 or 13 or 14 |
| 16 | cardiovascular diseases/ or exp heart diseases/ or exp vascular diseases/ |
| 17 | (myocardial or cardiovascular or cardiac or ((chronic or "long-term" or "long term" or longterm or lifelong or "life long" or "life-long" or permanent or persisting) adj3 heart*1)).tw,kf. |
| 18 | 16 or 17 |
| 19 | exp Diabetes Mellitus/ |
| 20 | (diabetes or diabetic).tw,kf. |
| 21 | 19 or 20 |
| 22 | exp Chronic Disease/ or Noncommunicable Diseases/ |
| 23 | ((chronic or "long-term" or "long term" or longterm or lifelong or "life long" or "life-long" or permanent or persisting) adj3 (illness* or disease* or condition* or diagnos* or disorder*)).tw,kf. |
| 24 | (NCD* or ((noncommunicable or "non communicable" or "non-communicable" or "non-infectious" or "non infectious" or noninfectious) adj2 disease*)).tw,kf. |
| 25 | 22 or 23 or 24 |
| 26 | 6 or 15 or 18 or 21 or 25 |
| 27 | 3 and 26 |
| 28 | Randomized controlled trials as Topic/ or Randomized controlled trial/ or Random allocation/ or Double blind method/ or Single blind method/ or Clinical trial/ or exp Clinical Trials as Topic/ or evaluation study/ or Placebos/ |
| 29 | ((clinic* or control*3) adj trial*).mp,pt. or random*.mp. or rct*.tw,kf. |
| 30 | (placebo* or ((singl* or doubl* or treb* or tripl*) adj (blind*3 or mask*3))).tw. |
| 31 | 28 or 29 or 30 |
| 32 | 27 and 31 |
| 33 | limit 32 to (address or autobiography or editorial or historical article or letter or periodical index or personal narrative or portrait) |
| 34 | 32 not 33 |
| 35 | limit 34 to yr="2008 -Current" |

| Database(s): **Embase**1974 to 2020 March 24 | |
| --- | --- |
| Search Strategy: | |
| **#** | **Searches** |
| 1 | empowerment/ |
| 2 | empower*.tw,kw. |
| 3 | 1 or 2 |
| 4 | exp cancer survival/ or Cancer Survivor/ or (Survivor/ and (Cancer Patient/ or exp Neoplasm/ or cancer.tw,kw.)) or ((cancer or neoplasm*) and surviv*).tw,kw. |
| 5 | cancer fatigue/ or ((Fatigue/ or fatigue*.tw,kw.) and (exp Neoplasms/ or (cancer or neoplasm*).tw,kw.)) |
| 6 | 4 or 5 |
| 7 | exp obstructive airway disease/ |
| 8 | (chronic or "long-term" or "long term" or longterm or lifelong or "life long" or "life-long" or permanent or persisting).tw,kw. |
| 9 | 7 and 8 |
| 10 | exp chronic obstructive lung disease/ |
| 11 | exp asthma/ |
| 12 | (((chronic or "long-term" or "long term" or longterm or lifelong or "life long" or "life-long" or permanent or persisting) adj3 (pulmonary or lung*1 or airway* or airflow* or bronch* or respirat*)) or emphysema*).tw,kw. |
| 13 | (COPD or COAD or COBD or AECB).tw,kw. |
| 14 | (asthma*1 or asthmatic).tw,kw. |
| 15 | 9 or 10 or 11 or 12 or 13 or 14 |
| 16 | cardiovascular disease/ or exp heart disease/ or exp vascular disease/ |
| 17 | (myocardial or cardiovascular or cardiac or ((chronic or "long-term" or "long term" or longterm or lifelong or "life long" or "life-long" or permanent or persisting) adj3 heart*1)).tw,kw. |
| 18 | 16 or 17 |
| 19 | exp diabetes mellitus/ |
| 20 | (diabetes or diabetic).tw,kw. |
| 21 | 19 or 20 |
| 22 | exp chronic disease/ or non communicable disease/ |
| 23 | ((chronic or "long-term" or "long term" or longterm or lifelong or "life long" or "life-long" or permanent or persisting) adj3 (illness* or disease* or condition* or diagnos* or disorder*)).tw,kw. |
| 24 | (NCD* or ((noncommunicable or "non communicable" or "non-communicable" or "non-infectious" or "non infectious" or noninfectious) adj2 disease*)).tw,kw. |
| 25 | 22 or 23 or 24 |
| 26 | 6 or 15 or 18 or 21 or 25 |
| 27 | 3 and 26 |
| 28 | "randomized controlled trial (topic)"/ or exp randomized controlled trial/ or randomization/ or double blind procedure/ or single blind procedure/ or exp clinical trial/ or exp "clinical trial (topic)"/ or evaluation study/ or placebo/ |
| 29 | ((clinic* or control*3) adj trial*).mp,pt. or random*.mp. or rct*.tw,kw. |
| 30 | (placebo* or ((singl* or doubl* or treb* or tripl*) adj (blind*3 or mask*3))).tw. |
| 31 | 28 or 29 or 30 |
| 32 | 27 and 31 |
| 33 | limit 32 to (editorial or letter) |
| 34 | 32 not 33 |

| Database(s): **APA PsycInfo**1806 to March Week 3 2020 | | | |
| --- | --- | --- | --- |
| Search Strategy: | |  | |
| **#** | | **Searches** | |
| 1 | | Empowerment/ | |
| 2 | | empower*.tw. | |
| 3 | | 1 or 2 | |
| 4 | | (Survivors/ and (exp Neoplasms/ or cancer.tw.)) or ((cancer or neoplasm*) and surviv*).tw. | |
| 5 | | (fatigue/ or fatigue*.tw.) and (exp Neoplasms/ or cancer.tw. or neoplasm*.tw.) | |
| 6 | | 4 or 5 | |
| 7 | | exp Lung Disorders/ | |
| 8 | | (chronic or "long-term" or "long term" or longterm or lifelong or "life long" or "life-long" or permanent or persisting).tw. | |
| 9 | | 7 and 8 | |
| 10 | | exp Chronic Obstructive Pulmonary Disease/ | |
| 11 | | exp Asthma/ | |
| 12 | | (((chronic or "long-term" or "long term" or longterm or lifelong or "life long" or "life-long" or permanent or persisting) adj3 (pulmonary or lung*1 or airway* or airflow* or bronch* or respirat*)) or emphysema*).tw. | |
| 13 | | (COPD or COAD or COBD or AECB).tw. | |
| 14 | | (asthma*1 or asthmatic).tw. | |
| 15 | | 9 or 10 or 11 or 12 or 13 or 14 | |
| 16 | | exp Cardiovascular Disorders/ | |
| 17 | | (myocardial or cardiovascular or cardiac or ((chronic or "long-term" or "long term" or longterm or lifelong or "life long" or "life-long" or permanent or persisting) adj3 heart*1)).tw. | |
| 18 | | 16 or 17 | |
| 19 | | exp diabetes/ | |
| 20 | | (diabetes or diabetic).tw. | |
| 21 | | 19 or 20 | |
| 22 | | chronic illness/ | |
| 23 | | ((chronic or "long-term" or "long term" or longterm or lifelong or "life long" or "life-long" or permanent or persisting) adj3 (illness* or disease* or condition* or diagnos* or disorder*)).tw. | |
| 24 | | (NCD* or ((noncommunicable or "non communicable" or "non-communicable" or "non-infectious" or "non infectious" or noninfectious) adj2 disease*)).tw. | |
| 25 | | 22 or 23 or 24 | |
| 26 | | 6 or 15 or 18 or 21 or 25 | |
| 27 | | 3 and 26 | |
| 28 | | exp Clinical Trials/ or exp treatment effectiveness evaluation/ or random sampling/ or placebo/ or quantitative methods/ or Experimental Methods/ or Quasi Experimental Methods/ | |
| 29 | | (random* or rct* or ((clinic* or control*3) adj trial*)).tw. | |
| 30 | | (placebo* or ((singl* or doubl* or treb* or tripl*) adj (blind*3 or mask*3))).tw. | |
| 31 | | 28 or 29 or 30 | |
| 32 | | 27 and 31 | |
| 33 | | limit 32 to yr="2008 -Current" | |
| 35 | limit 34 to yr="2008 -Current" | |  |

| **CINAHL with Full Text** | |
| --- | --- |
|  |  |
| **Print Search History** | |
|  | Wednesday, March 25, 2020 9:44:11 AM |
| **#** | **Query** |
| S33 | S27 AND S31 |
| S32 | S27 AND S31 |
| S31 | S28 OR S29 OR S30 |
| S30 | (placebo* or ((singl* or doubl* or treb* or tripl*) N0 (blind* or mask*))) |
| S29 | ((clinic* or control*) N0 trial*) or random* or rct* |
| S28 | (MH "Experimental Studies+") OR (MH "Quasi-Experimental Studies+") OR (MH "Random Sample+") OR (MH "Evaluation Research+") OR (MH "Placebos") OR (MH "Placebo Effect") |
| S27 | S3 AND S26 |
| S26 | S6 OR S15 OR S18 OR S21 OR S25 |
| S25 | S22 OR S23 OR S24 |
| S24 | (NCD* or ((noncommunicable or "non communicable" or "non-communicable" or "non-infectious" or "non infectious" or noninfectious) N1 disease*)) |
| S23 | ((chronic or "long-term" or "long term" or longterm or lifelong or "life long" or "life-long" or permanent or persisting) N2 (illness* or disease* or condition* or diagnos* or disorder*)) |
| S22 | (MH "Chronic Disease+") or (MH "Noncommunicable Diseases") |
| S21 | S19 OR S20 |
| S20 | (diabetes or diabetic) |
| S19 | (MH "Diabetes Mellitus+") |
| S18 | S16 OR S17 |
| S17 | (myocardial or cardiovascular or cardiac or ((chronic or "long-term" or "long term" or longterm or lifelong or "life long" or "life-long" or permanent or persisting) N2 heart#)) |
| S16 | (MH "Cardiovascular Diseases") OR (MH "Vascular Diseases+") OR (MH "Heart Diseases+") |
| S15 | S9 OR S10 OR S11 OR S12 OR S13 OR S14 |
| S14 | (asthma# or asthmatic) |
| S13 | (COPD or COAD or COBD or AECB) |
| S12 | ((chronic or "long-term" or "long term" or longterm or lifelong or "life long" or "life-long" or permanent or persisting) N2 (pulmonary or lung# or airway* or airflow* or bronch* or respirat*)) or emphysema* |
| S11 | (MH "Asthma+") |
| S10 | (MH "Pulmonary Disease, Chronic Obstructive+") |
| S9 | S7 AND S8 |
| S8 | chronic or "long-term" or "long term" or longterm or lifelong or "life long" or "life-long" or permanent or persisting |
| S7 | (MH "Lung Diseases, Obstructive+") |
| S6 | S4 OR S5 |
| S5 | MH "Cancer Fatigue" OR ((MH "Fatigue" or fatigue*) AND (MH "Neoplasms+" OR cancer or neoplasm*) |
| S4 | (MH "Cancer Survivors") OR ((MH "Survivors" or surviv*) AND (MH "Cancer Patients" OR MH "Neoplasms+" OR cancer or neoplasm*) |
| S3 | S1 OR S2 |
| S2 | empower* |
| S1 | (MH "Empowerment") |

| Cochrane Central | |
| --- | --- |
| Date Run: | 25.03.2020 15:08 |
| Comment: |  |
|  |  |
| ID | Search |
| #1 | (empower*):ti,ab,kw |
| #2 | MeSH descriptor: [Power, Psychological] explode all trees |
| #3 | #1 or #2 |
| #4 | ((cancer or neoplasm*) and surviv*):ti,ab,kw |
| #5 | ((cancer or neoplasm*) and fatigue*):ti,ab,kw |
| #6 | #4 or #5 |
| #7 | MeSH descriptor: [Lung Diseases, Obstructive] explode all trees |
| #8 | ((chronic or "long-term" or "long term" or longterm or lifelong or "life long" or "life-long" or permanent or persisting)):ti,ab,kw |
| #9 | #7 and #8 |
| #10 | MeSH descriptor: [Pulmonary Disease, Chronic Obstructive] explode all trees |
| #11 | MeSH descriptor: [Asthma] explode all trees |
| #12 | (emphysema* or ((chronic or "long-term" or "long term" or longterm or lifelong or "life long" or "life-long" or permanent or persisting) NEAR/2 (pulmonary or lung? or airway* or airflow* or bronch* or respirat*))):ti,ab,kw |
| #13 | (COPD or COAD or COBD or AECB):ti,ab,kw |
| #14 | (asthma? or asthmatic):ti,ab,kw |
| #15 | {OR #9-#14} |
| #16 | MeSH descriptor: [Cardiovascular Diseases] this term only |
| #17 | MeSH descriptor: [Heart Diseases] explode all trees |
| #18 | MeSH descriptor: [Vascular Diseases] explode all trees |
| #19 | ((myocardial or cardiovascular or cardiac) or ((chronic or "long-term" or "long term" or longterm or lifelong or "life long" or "life-long" or permanent or persisting) NEAR/2 heart?)):ti,ab,kw |
| #20 | {OR #16-#19} |
| #21 | MeSH descriptor: [Diabetes Mellitus] explode all trees |
| #22 | (diabetes or diabetic):ti,ab,kw |
| #23 | #21 or #22 |
| #24 | MeSH descriptor: [Chronic Disease] explode all trees |
| #25 | MeSH descriptor: [Noncommunicable Diseases] this term only |
| #26 | ((chronic or "long-term" or "long term" or longterm or lifelong or "life long" or "life-long" or permanent or persisting) NEAR/2 (illness* or disease* or condition* or diagnos* or disorder*)):ti,ab,kw |
| #27 | (NCD* or ((noncommunicable or "non communicable" or "non-communicable" or "non-infectious" or "non infectious" or noninfectious) NEAR/1 disease*)):ti,ab,kw |
| #28 | {OR #24-#27} |
| #29 | #6 or #15 or #20 or #23 or #28 |
| #30 | #3 and #29 with Publication Year from 2008 to present, in Trials |

Web of Science


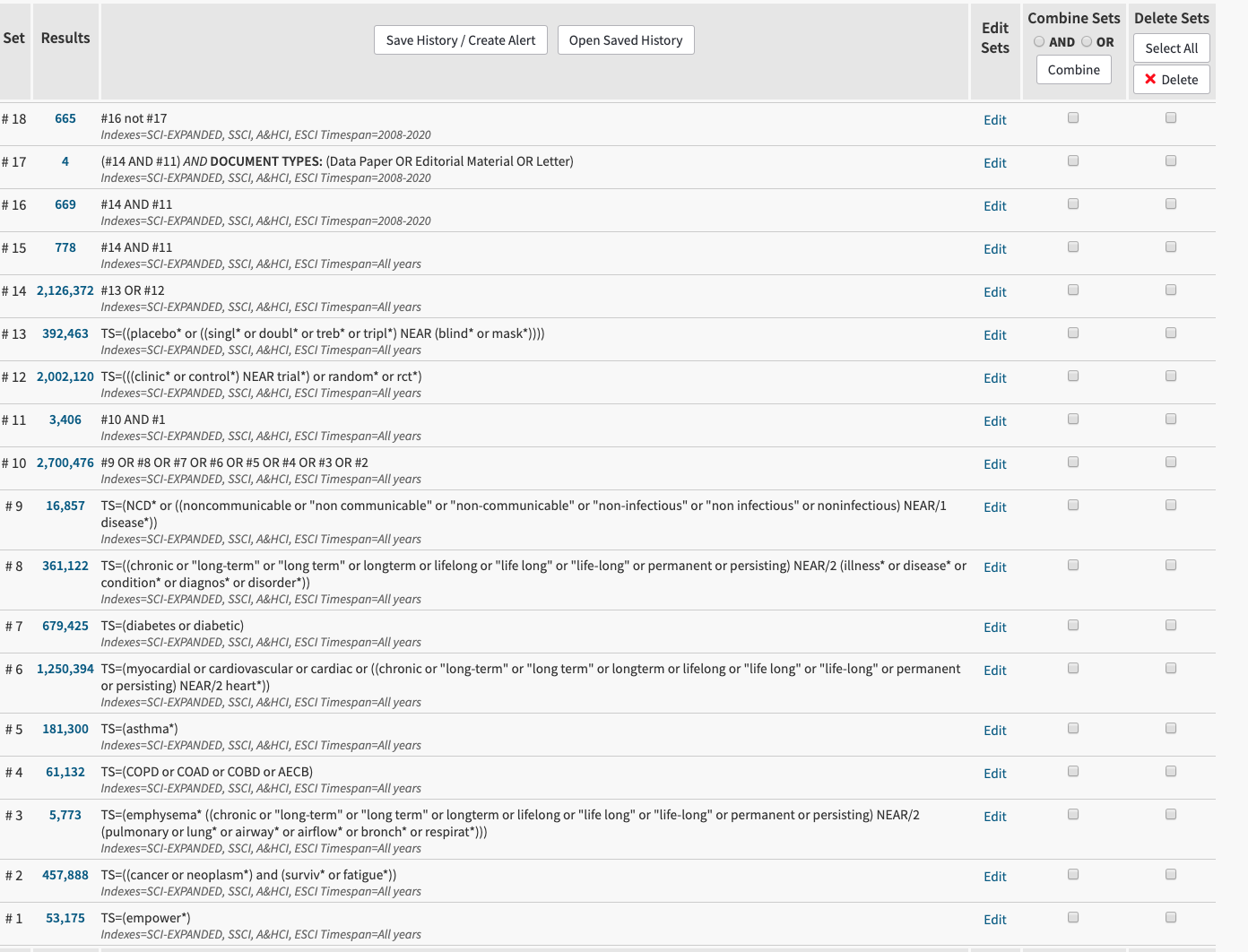

Supplement: Supplementary file 1 — Additional file 1: Supplementary file 1. Literature Search Strategies from Ovid Medline, Embase, Cinahl, APA PsycInfo, Cochrane Central and Web of Science. [file 12913_2023_9895_MOESM1_ESM.docx]
