# Supplementary material for: Empowerment interventions designed for persons living with chronic disease - a systematic review and meta-analysis of the components and efficacy of format on patient-reported outcomes
Source: BMC Health Serv Res. 2023 Aug 25;23:911. doi: 10.1186/s12913-023-09895-6 (PMC10463815; doi:10.1186/s12913-023-09895-6)
Supplement: Supplementary file 2 — Additional file 2: Supplementary file 2. Table 5. Risk of Bias (ROB) assessments and short reasons for the selected assessments. [file 12913_2023_9895_MOESM2_ESM.docx]

Supplementary file 2:

Table 5. Risk of Bias (ROB) assessments and short reasons for the selected assessments

| ROB | **Domain 1:** Risk of bias arising from the randomization process | **Domain 2:** Risk of bias due to deviations from the intended interventions (effect of assignment to intervention) | **Domain 3:** Missing outcome data | **Domain 4:** Risk of bias in measurement of the outcome | **Domain 5**: Risk of bias in selection of the reported result | **Overall risk of bias** |
| --- | --- | --- | --- | --- | --- | --- |
| **Admiraal et al (2017)** | **Low** | **Some concern**  **(no blinding)** | **Low** | **High**  **(Outcome assessors aware)** | **Low** | **High** |
| **Lenjawi et al (2017)** | **Low** | **Some concern**  **(Deviations from the trial context and no blinding)** | **Low** | **Low** | **Low** | **Some concerns** |
| **Moein et al (2017)** | **Low** | **Some concern**  **(No info on blinding)** | **Some concern** | **High**  **(Knowledge of the assigned intervention could influence participant-reported outcomes)** | **Low** | **High** |
| **Üzar-Özcetin et al (2018)** | **Low** | **Some concerns**  **(Careers and people delivering the interventions aware)** | **Low** | **High**  **(Proms and no blinding)** | **Low** | **High** |
| **Tabari et al (2018)** | **Some concerns**  **(not randomized)** | **High**  **(no intention to treat info on missing/ not randomized)** | **Low** | **High**  **No blinding** | **High**  (only mean score) | High |
| **Visser et al (2018)** | **Low** | **High**  **(not blinded for randomization)** | **Low** | **Some concerns (Knowledge of the assigned intervention could influence participant-reported outcomes)** | **Low** | High |
| **Aslani et al (2019)** | **Low** | **Some concern**  **(Pat aware of assigned intervention)** | **Low** | **High**  **(no blinding and researcher in all roles)** | **Low** | **High** |
| **Cheng et al (2018)** | **Low** | **Low** | **Low** | **Low** | **Low** | **Low** |
| **Dehghan et al (2018)** | **Low** | **High (no intention to treat)** | **High (lack of information and missing outcome data )** | **High**  **(no blinding and PROMS)** | **Low** | **High** |
| **Almeida et al (2019)** | **Some concerns**  **(no information about randomization)** | **Some concerns**  **(no blinding)** | **Some concerns (High drop out and no intention to treat)** | **High**  **(outcome assessors aware)** | **Low** | **High** |
| **Cheng et al (2019)** | **Low** | **Low** | **Low** | **Low** | **Low** | **Low** |
| **Deghan et al (2017)** | **High**  **pre/post design)** | **Some concerns**  **(no info on blinding)** | **High (high drop out, loss of follow up, no int to treat)** | **Some concerns (Only Proms and pat knows the interv)** | **Low** | High |
| **Shin et al (2016)** | **High (no randomization (pre/post)** | **Some concerns (no blinding, no intention to treat)** | **Low** | **Low** | **Low** | High |
| **Fardazar et al (2018)** | **High (quasi rand)** | **High (no blinding, no intention to treat)** | **Low** | **High (knowledge of intervention received)** | **Low** | High |
| **Musavinasab et al (2016)** | **Low** | **Some concerns (no blinding and no intention to treat)** | **Low** | **High (Proms and pat are outcome assessors, no blinding)** | **Low** | High |
| **Sit et al (2016)** | **Low** | **Low** | **Low** | **Low** | **Low** | **Low** |
| **Azimi et al (2016)** | **Low** | **Low** | **Low** | **Low** | **Low** | **Low** |
| **Zamanzadeh et al (2017)** | **Low** | **Some concerns (pat aware of the intervention, proms)** | **Low** | **Some concerns (pat not blinded + proms (carers were blinded)** | **Low** | **Some concerns** |
| **Van Puffelen et al (2019)** | **Low** | **Low** | **Low** | **High (Control group got two extra questionnaires)** | **Low** | High |
| **Zoun et al (2019)** | **Low** | **Some concerns (participants aware, no blinding)** | **Low** | **Some concerns (pat aware and PROMS)** | **Some concerns (lack of information in the article)** | **Some concerns** |
