# Supplementary material for: Empowerment interventions designed for persons living with chronic disease - a systematic review and meta-analysis of the components and efficacy of format on patient-reported outcomes
Source: BMC Health Serv Res. 2023 Aug 25;23:911. doi: 10.1186/s12913-023-09895-6 (PMC10463815; doi:10.1186/s12913-023-09895-6)
Supplement: Supplementary file 3 — Additional file 3: Supplementary file 3. Graph Summary plot ROB2. [file 12913_2023_9895_MOESM3_ESM.docx]

Supplementary file 3 Graph Summary plot ROB2


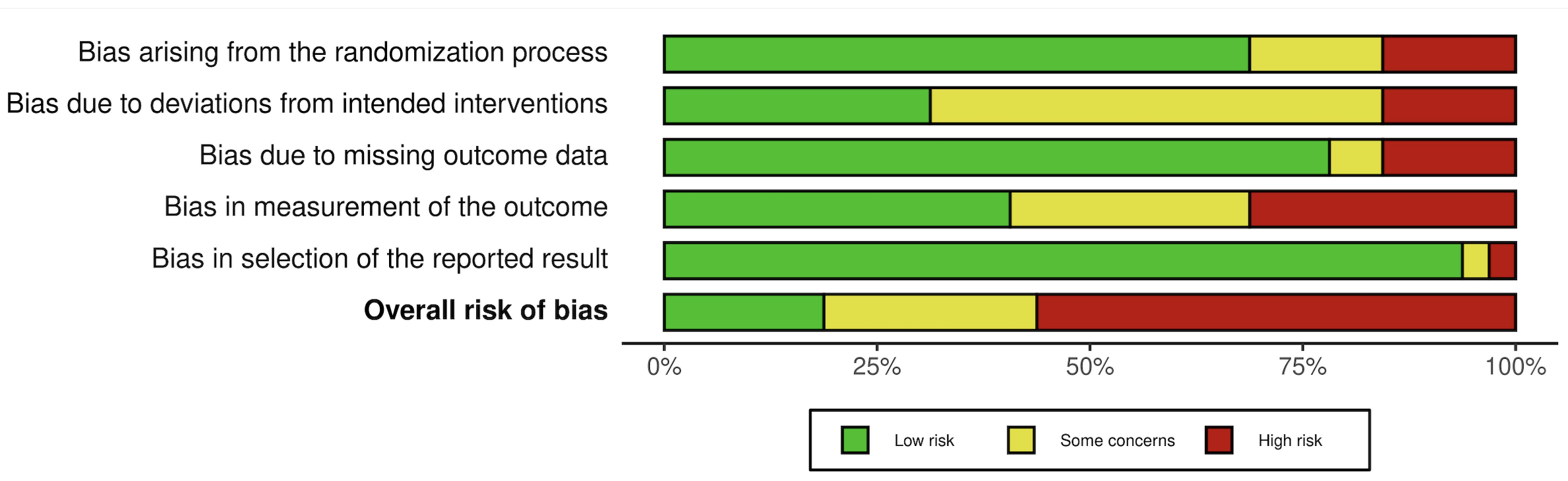


Risk of bias graph following the ROB2 guidelines of the Cochrane Library. Green indicates low risk of bias, yellow indicates unclear risk of bias, and red indicates high risk of bias.
